# High-resolution conditional MR image synthesis through the PACGAN framework

**DOI:** 10.1038/s41598-025-16257-1

**Published:** 2025-10-01

**Authors:** Matteo Lai, Chiara Marzi, Luca Citi, Stefano Diciotti

**Affiliations:** 1 Department of Electrical, Electronic, and Information Engineering “Guglielmo Marconi” - DEI, University of Bologna, Cesena, 47522 Italy; 2 Department of Statistics, Computer Science and Applications “Giuseppe Parenti”, University of Florence, Florence, Italy; 3 School of Computer Science and Electronic Engineering, University of Essex, Colchester, CO4 3SQ UK; 4 Alma Mater Research Institute for Human-Centered Artificial Intelligence, University of Bologna, Bologna, 40121 Italy

**Keywords:** Biomedical engineering, Computational science

## Abstract

Deep learning algorithms trained on medical images often encounter limited data availability, leading to overfitting and imbalanced datasets. Synthetic datasets can address these challenges by providing a priori control over dataset size and balance. In this study, we present PACGAN (Progressive Auxiliary Classifier Generative Adversarial Network), a proof-of-concept framework that effectively combines Progressive Growing GAN and Auxiliary Classifier GAN (ACGAN) to generate high-quality, class-specific synthetic medical images. PACGAN leverages latent space information to perform conditional synthesis of high-resolution brain magnetic resonance (MR) images, specifically targeting Alzheimer’s disease patients and healthy controls. Trained on the Alzheimer’s Disease Neuroimaging Initiative (ADNI) dataset, PACGAN demonstrates its ability to generate realistic synthetic images, which are assessed for quality using quantitative metrics. The ability of the generator to perform proper target synthesis of the two classes was also assessed by evaluating the performance of the pre-trained discriminator when classifying real unseen images, which achieved an area under the receiver operating characteristic curve (AUC) of 0.813, supporting the ability of the model to capture the target characteristics of each class. The pre-trained models of the generator and discriminator, together with the source code, are available in our repository: https://github.com/aiformedresearch/PACGAN.

**Supplementary Information:**

The online version contains supplementary material available at 10.1038/s41598-025-16257-1.

## Introduction

The development of deep learning (DL) techniques, with their ability to automatically extract high-level features from raw data, shows great promise in medical imaging, where data are inherently digital. Convolutional neural networks (CNNs)^1^ are the most common type of DL models for medical imaging and could potentially automate various time-consuming tasks performed by radiologists for disease diagnosis^2^. These algorithms can assess features that were previously overlooked by radiologists and arrive at a repeatable conclusion in a fraction of the time required for a human interpreter. Although DL techniques can provide such benefits to radiologists, a CNN has many training parameters that can reach millions, which requires a large amount of training data to avoid overfitting. However, medical images typically have a dataset size lower by orders of magnitude compared to natural images due to limited availability caused by data privacy regulations^3^. Furthermore, medical data are generally imbalanced because healthy subjects usually outnumber patients with a disease. Since classification algorithms typically rely on balanced datasets, they may encounter varying degrees of defects when dealing with imbalanced data classification because the model may interpret data contained in the smaller classes as noisy values.

Data augmentation is a consolidated approach to address these issues by modifying the training data. It provides a way to artificially expand a dataset to help the model avoid learning features that are too specific to the original training data, which enhances the model’s generalizability and ultimately improves the performance on the test set. Basic augmentation methods employ geometrical transformations such as translation, rotation, flipping, cropping, and occlusion. While they are simple to compute, their output is strongly dependent on the original data^4–6^.

A promising and increasingly popular technique in artificial intelligence is the generation of synthetic data. Among the generative models, two techniques have emerged in recent literature:*generative adversarial networks* (GANs)^7^ and *diffusion models*^8^. GANs are a DL-based approach that can automatically learn to generate realistic images belonging to the same distribution as the training set, achieved through the adversarial training of two DL models. In contrast, diffusion models iteratively denoise noisy images through a learned diffusion process to generate new data instances aligned with the training data distribution. Despite the promise of diffusion models, recent studies have highlighted concerns regarding their tendency to overfit training data compared to GANs^9,10^. This propensity for overfitting is a serious limitation in sensitive domains such as healthcare, where securing patient privacy is critical.

This characteristic makes GANs particularly well-suited for generating medical images, leading the research community to increasingly employ GANs in the field of medical imaging^11–13^. One of the main potential benefits of GANs is that they take many decisions away from the user. An ideal GAN is able to estimate the underlying theoretical distribution of the finite training samples, and thereby the effect is to simultaneously apply augmentation to each source of variance within the dataset. While traditional methods can only augment or remove linear sources of variance, such as size or rotation, a trained GAN can directly learn the distribution of many sources of variance from the available data. For example, when training a GAN with a dataset of MRI brain images with different gyro-sulcal morphology, the model can learn the source of variance of the gyri and sulci shapes, enabling the synthesis of images along the continuum of all shapes. As a result, GANs infer this model directly from the training data without the need for a complex model of realistic gyro-sulcal morphology. One potential limitation of using GANs is the possibility of producing images with imperfect fidelity. However, realism is not necessarily a goal when creating augmented data because even unrealistic images can help produce a more generalizable model^5^.

During the training phase, two DL models – a generator and a discriminator – are jointly trained in a zero-sum game, whereby as the discriminator improves its ability to differentiate between real and fake samples, it reduces the generator’s performance in producing realistic synthetic samples, and vice versa. The discriminator is a classifier trained to distinguish real images from fake ones – produced by the generator. The generator takes a random vector $\:z$ as input and is trained to generate synthetic images that are realistic according to the discriminator output, treating the input vector as its latent space. However, a drawback of GANs is the lack of control over the modes of the generated data. While they are capable of generating new random plausible examples for a given dataset, there is no way to control the types of generated data. In scenarios where datasets include additional information, such as a class label, it is desirable to use that to synthesize target data. The *Auxiliary Classifier GAN* (ACGAN)^14^ modifies the GAN architecture to generate target images. The generator of the ACGAN takes as input both the vector $\:z$ and the label $\:y$ of the data to be generated and produces synthetic images of the target class. The discriminator, on the other hand, exclusively receives images (real and generated) and is trained not only to determine their realness but also to estimate their class label. In this way, the ACGAN overcomes the aforementioned drawback enabling targeted image synthesis. Another limitation of both GAN and ACGAN is the limited resolution of the generated images. The generator must learn how to output both the large-scale structure and fine details simultaneously, leading to a poor capacity for generating high-resolution images. Moreover, the details of the high-resolution images make it easier to discriminate real images from fake ones, causing the training process to fail. Finally, the training of a GAN that generates large images, such as 1024 $\:\times\:$ 1024, requires smaller minibatches, leading to greater instability in the training process^15^. The *Progressive Growing GAN*^15^ proposes a solution to the problem of training stable GAN models for larger images. The basic idea is to train the GAN network in multiple phases, incrementally adding layers to the discriminator and generator models to manage images with increasingly higher resolution. This approach enables the model to first discover the large-scale structure of the image distribution and then shift its focus to increasingly finer-scale details, instead of having to learn all scales simultaneously. Although it can generate high-resolution images, the Progressive Growing GAN has the same drawback as GANs, i.e., it cannot perform conditional image synthesis.

In this study, we introduce *Progressive ACGAN* (PACGAN), a proof-of-concept model that integrates the strengths of ACGAN and progressive growing GAN to generate high-resolution, class-specific medical images. PACGAN is designed to address the dual challenge of realistic high-resolution synthesis and controlled class-conditioned generation, making it a promising candidate for medical data augmentation. PACGAN consists of a generator that synthesizes high-resolution brain MR images conditioned on class labels and a discriminator that distinguishes real from synthetic images while also classifying them into categories. By evaluating the pre-trained discriminator on unseen real images, we assessed whether the learned latent representations are sufficiently robust to guide the generation of high-quality, class-specific synthetic images. To demonstrate its capability, we trained and tested PACGAN on 2444 structural magnetic resonance (MR) images from the Alzheimer’s Disease Neuroimaging Initiative (ADNI)^16^ dataset (http://adni.loni.usc.edu), which includes MRI data of patients with Alzheimer’s Disease (AD) and healthy controls (HC), and we used it to generate and classify images of the two classes.

To further assess the practical utility of PACGAN, we explored its application in a downstream classification task involving the diagnosis of AD from brain MR images. Specifically, we simulated a realistic scenario in which a researcher has access only to a small labeled dataset, a common situation due to privacy regulations or restrictive data sharing agreements. In such low-data settings, training a classifier from scratch is challenging, and using a pretrained model can be highly beneficial – especially if the pretraining was performed on images from the same domain. However, when access to large sets of real images for pretraining is not possible, synthetic images generated by a model like PACGAN may serve as an alternative. Therefore, we tested whether pretraining a ResNet-18 classifier on PACGAN-generated images could enhance classification performance in this constrained setting, thereby evaluating the value of PACGAN as a tool to support learning when real data is scarce or inaccessible.

## Results

The quality of the images generated by PACGAN was quantitatively evaluated using three metrics: Fréchet Inception Distance (FID)^17,18^, Kernel Inception Distance (KID)^18,19^, and Structural Similarity Index Measure (SSIM)^20^. These metrics assess different aspects of similarity between the generated and real images, providing a multifaceted evaluation of the performance of PACGAN. While FID and KID quantify the distance between feature distributions, assessing the distributional and statistical properties of the generated images, SSIM focuses on pixel-level structural similarity, capturing fine-grained details that reflect local image quality.

The computed metric values are presented in Table 1, where they were calculated under three different configurations. In the first row (Real/Fake), the synthetic images were compared to real ones to assess their realness. In the subsequent rows, the metrics were computed by considering images from the same distribution (Real/Real and Fake/Fake) to estimate their diversity. The comparison between the last two values was used to evaluate the network’s capability to capture the real distribution.

**Table 1:** Synthesis performance of the PACGAN for the final model trained on the entire training set, computed considering the images of the test set as the “real” distribution. The quality of the synthetic images was evaluated using the Fréchet inception distance (FID), kernel inception distance (KID), and structural similarity index measure (SSIM). The range of each metric is indicated in the header. The symbol $\:\uparrow\:$ indicates that a higher value corresponds to greater similarity, while $\:\downarrow\:$ means the opposite. The quality metrics computed for the comparison between real and synthetic images (Real/Fake) serve as an index of the realness of the synthetic images. The same metrics computed between images from the same distribution (Real/Real and Fake/Fake) enable the assessment of the diversity of the generated images. Image data IDs for real images used in the computation are provided in supplementary methods, and synthetic images are accessible as a NIfTI file in the supplementary material.

	FID $\:\downarrow\:$	KID $\:\downarrow\:$	SSIM $\:\uparrow\:$
	$\:[0,{\infty\:})$	$\:[0,{\infty\:})$	$\:[0,1]$
Real/fake	51.894	0.052 $\:\pm\:$ 0.005	0.602 $\:\pm\:$ 0.037
Real/real	0.000	0.000 $\:\pm\:$ 0.001	0.611 $\:\pm\:$ 0.065
Fake/fake	0.000	0.000 $\:\pm\:$ 0.002	0.621 $\:\pm\:$ 0.056

**Fig. 1:**
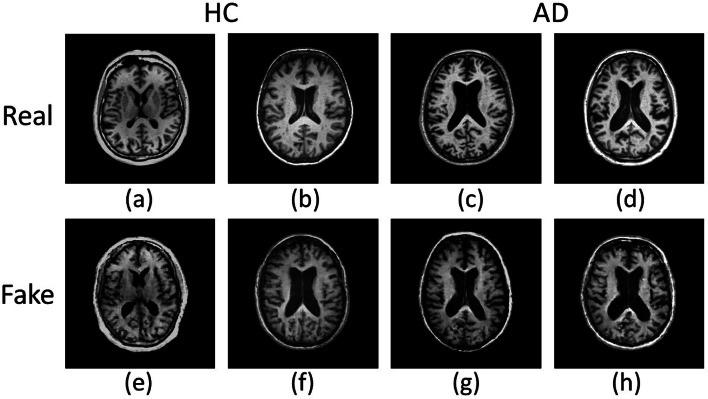
Comparison of synthetic images generated by the PACGAN with real brain MRI. (**a**, **b**) display real brain MRI of healthy controls (HC), while (**c**, **d**) depict real brain MRI images of Alzheimer’s Disease (AD) subjects. Image Data IDs from the ADNI dataset: I217939, I1391580, I50702, I1190066, respectively. On the lower row, (**e**, **f**) present synthetic brain MRI images of HC subjects, while (**g**, **h**) show synthetic brain MRI images of AD subjects.

To assess the capability of PACGAN in generating target images, we computed the KID and the SSIM for images within the same class. For KID, we used batches of 50 images, while for SSIM, we compared 1000 image pairs, as detailed in the *Methods* section. Table 2 presents the mean and standard deviation of these two metrics for each class. The similarity between synthetic and real images (Real/Fake) within each class should reflect the inherent balance observed in the real dataset (Real/Real). Furthermore, the distribution of synthetic data (Fake/Fake) should exhibit similar behavior. Figure 1 shows representative examples of synthetic images generated by PACGAN for each class along with images from the test set.

**Table 2:** Mean and standard deviation of the kernel inception distance (KID) and structural similarity index measure (SSIM) for each class: healthy controls (HC) and alzheimer’s disease (AD). The range of each metric is indicated in the table header. The symbol $\:\uparrow\:$ means that a higher value indicates greater similarity, while $\:\downarrow\:$ denotes the opposite. The KID was computed using batches of 50 images from the same class, while the SSIM was calculated between 1000 couples of images from the same class. Supplementary methods contain image data IDs for real images involved in the computation, and synthetic images are available in the supplementary material as a NIfTI file.

	HC		AD	
	KID $\:\downarrow\:$	SSIM $\:\uparrow\:$	KID $\:\downarrow\:$	SSIM $\:\uparrow\:$
	$\:[0,{\infty\:})$	$\:[0,1]$	$\:[0,{\infty\:})$	$\:[0,1]$
Real/fake	0.050 $\:\pm\:$ 0.004	0.598 $\:\pm\:$ 0.036	0.059 $\:\pm\:$ 0.005	0.606 $\:\pm\:$ 0.037
Real/real	0.000 $\:\pm\:$ 0.001	0.605 $\:\pm\:$ 0.055	0.000 $\:\pm\:$ 0.001	0.618 $\:\pm\:$ 0.074
Fake/fake	0.000 $\:\pm\:$ 0.001	0.612 $\:\pm\:$ 0.049	0.000 $\:\pm\:$ 0.001	0.629 $\:\pm\:$ 0.061

To further evaluate the performance of PACGAN in generating target images, we assessed the ability of the trained discriminator to classify new real instances. Since the generator was trained using the discriminator as a guide, this evaluation provides an indirect measure of the generator’s capability to synthesize target images. The area under the receiver operating characteristic curve (AUC) was 0.814 for real images (on a separate test set, different from PACGAN’s training set) and 1.0 for synthetic ones. To contextualize the performance of the discriminator, we trained several ResNet models (ResNet-18, ResNet-34, ResNet-50, and ResNet-101) using the same training set as PACGAN and evaluated them on the same independent test set. These models, fine-tuned for 10 epochs using Adam optimizer with a learning rate of 0.001, achieved AUC scores of 0.760, 0.784, 0.755, and 0.748, respectively – all of which were lower than the 0.814 AUC achieved by the PACGAN discriminator.

To evaluate PACGAN’s utility in real-world scenarios, we simulated the situation of a researcher with limited access to labeled data attempting to improve classification performance through pretraining. Specifically, we compared two ResNet-18 classifiers: one pretrained on ImageNet, and the other pretrained on 3,000 synthetic brain MR images generated by PACGAN. Both models were then fine-tuned for 10 epochs on two subsets of real brain MR data, containing either 100 or 200 real images. Performance was evaluated on a separate test set of 100 images. Table 3 presents the AUC and coefficients of variation, computed over 100 bootstrap repetitions.

**Table 3:** Utility assessment. Average area under the ROC curve (AUC) and coefficient of variation (CV) computed for two ResNet-18 classifiers pretrained on imagenet and on PACGAN-generated images. Both models were fine-tuned using datasets of 100 and 200 images, respectively, across 100 bootstrap repetitions, and tested on a test set of 100 images.

Training set size	Average AUC		CV	
	ImageNet	PACGAN	ImageNet	PACGAN
100 images	0.678	0.703	0.1137	0.0727
200 images	0.713	0.760	0.0840	0.0533

The results presented in this study were obtained using a model that underwent a grid search to optimize three hyperparameters: $\:{z}_{dim}$, $\:em{b}_{dim}$, and $\:{\lambda\:}_{cls}$, as defined in the *Method* section. The process for selecting the best model is described in detail in the *PACGAN hyperparameter tuning* section below. Supplementary Table 1 provides a summary of the various hyperparameter combinations explored in the grid search ($\:{z}_{dim}\in\:\{100,\:300,\:512\}$, $\:em{b}_{dim}\in\:\{2,\:3,\:4,\:5,\:6,\:7,\:8\}$, $\:{\lambda\:}_{cls}\in\:\{3,\:4,\:5,\:6\}$) along with the corresponding results for each training configuration. For each training iteration, quantitative metrics (FID, KID, SSIM) were computed by comparing the synthetic images with real images (Real/Fake column) and with other synthetic images within the fake distribution itself (Fake/Fake column). Additionally, the AUC on the validation set was calculated. Based on these results, the hyperparameter configuration yielding the best performance was determined to be $\:{z}_{dim}=512$, $\:em{b}_{dim}=3$, and $\:{\lambda\:}_{cls}=4$, as detailed in the *PACGAN hyperparameter tuning* section. The results presented in Supplementary Table 1 demonstrate the high stability of PACGAN training, with only 2 out of 105 training runs failing to reach convergence. This indicates that the training process achieved convergence in 98.1% of the cases, consistently demonstrating excellent performance in image generation and classification of new instances. Supplementary Figs. 1–3 depict the average classification performance of the discriminator under varying values of the three hyperparameters. The consistent performance observed across all values indicates that the discriminator’s ability to differentiate between the classes remains unaffected by the hyperparameters of the generator.

## Discussion

This study introduces PACGAN, an adaptation of GAN-based architectures specifically developed to generate high-resolution synthetic images of a target class while monitoring the accuracy of class representation. The model achieves this by progressively training on labeled data of increasing size. In this study, we used a dataset comprised of 2D brain MRI images extracted from the central axial slice of each volume sourced from the ADNI dataset. The ADNI dataset encompasses images of both AD patients and HC. The training process involves two distinct networks, namely the generator and discriminator, which are trained in an adversarial manner. The objective is to enable the generator to synthesize new images by leveraging the discriminator’s feedback as a guiding mechanism. In this study, the discriminator is trained to perform two key tasks. Firstly, it distinguishes between real and fake data thus providing valuable guidance to the generator for synthesizing more realistic images. Additionally, the discriminator is also trained to classify images into the AD and HC classes, further assisting the generator in producing accurate representations of each class. Upon completion of the training process, the generator exhibits the capability to generate full-resolution synthetic brain MRI images of both AD patients and HC. At the same time, the discriminator demonstrates good accuracy in classifying new data instances, suggesting that its internal model, which guided the generator in synthesizing target images for both classes, accurately represents brain MRI slices of both HC and AD subjects. The quality of the synthetic images is deemed satisfactory. This is supported by the metrics presented in Table 1 and the visual evidence depicted in Fig. 1. Indeed, the synthetic images closely resemble the structural characteristics of real images, albeit with certain limitations in capturing finer details. The generator is equally effective in synthesizing target images for both classes, as evidenced by the similarity loss values provided in Table 2. These values indicate that the realism of the images from both classes is comparable, as assessed by three different similarity scores. The fidelity of representation in the synthetic instances for each class relies on the discriminative capabilities of the discriminator. Indeed, throughout the training process, the discriminator indirectly guides the generative process, influencing the generator’s ability to produce accurate representations. This is confirmed by the discriminator’s classification of the synthetic images, achieving an AUC value of 1.0. This result indicates that the generator successfully captures the latent space of the discriminator and generates target images according to it.

To further evaluate the effectiveness of the discriminator, we compared its performance to ResNet classifiers trained on the same dataset used to train PACGAN. Specifically, ResNet-18, ResNet-34, ResNet-50, and ResNet-101 were trained on real brain MRIs and tested on the same independent test set. The discriminator outperformed all of them, achieving an AUC of 0.814. This suggests that the discriminator effectively learned meaningful feature representations for classifying real brain MR images.

We also compared the performance of the discriminator to the state-of-the-art classifiers from the literature. However, this comparison was complicated by the fact that, in this exploratory investigation, we focused on generating 2D images. This approach, in fact, imposed limitations on the discriminator’s capabilities, as it was only able to classify based on a single slice of brain MRI for each subject, rather than considering the entire 3D volume. Consequently, the performance of our model did not reach that of state-of-the-art classifiers, which achieved AUC scores exceeding 0.89. This discrepancy can be attributed to the fact that these classifiers utilized 3D-CNN^21–26^, enabling them to analyze the complete volume of brain MRI for each subject. Alternatively, some studies incorporated classic CNN architectures while considering multiple slices for each subject^27,28^ and, in some cases, integrating genetic or demographic features. Despite the disadvantage of relying on a single brain MRI slice, PACGAN demonstrated a commendable ability to capture the distribution of real data with good accuracy. Our findings exhibit a promising behavior that we anticipate can be enhanced by extending the network’s functionality to work with 3D data.

To appraise the quality of the synthetic images produced by the generator, we computed three quantitative metrics as mentioned above. The metrics employed were FID and KID, which compare the feature distributions of real and synthetic images, quantifying how closely the generated images resemble the real data in terms of high-level representations. Additionally, we employed SSIM to evaluate perceptual similarity, which evaluates image quality based on pixel-wise structure, luminance, and texture. SSIM is particularly relevant for medical imaging, such as brain MRIs, as it takes into account structural patterns.

The FID is a widely utilized metric for evaluating the quality of synthetic images^29^, which has shown to be consistent with human perceptual evaluation of medical images^30^. However, its application to synthetic brain MRI has been limited, with only a few studies^31–34^ implementing it in the context of synthetic brain MRI. The reported FID values in these studies ranged between 37.01 and 131.62. However, direct comparison of FID scores across different studies can be challenging since this metric depends on the number of images used in its computation^19^. To address this challenge, we also implemented the KID as an additional metric. Unlike FID, KID is not biased by the number of images considered^19^. However, we were unable to find prior studies that employed this metric for assessing the realism of synthetic imaging data in our specific domain, thus limiting our ability to make direct comparisons. Supplementary Figs. 1–3 show that both FID and KID exhibit similar trends when varying the value of each hyperparameter. Considering the typically limited availability of medical imaging data and the resulting potential bias of FID, we recommend using KID as an alternative for assessing the realism of synthetic images in the medical field. This is particularly relevant where the scarcity of images is a common challenge, as KID provides a more suitable evaluation metric. It is worth noting that both FID and KID rely on the Inception Net which was trained on ImageNet, a dataset containing natural images. As a result, discrepancies between these metrics and human judgment may arise when working with non-ImageNet data, such as brain MRI.

The primary metric commonly used to assess the realness of synthetic brain MRI is SSIM^35^. This score measures the similarity between pairs of images ranging from 0 to 1, with 1 indicating maximum similarity (i.e., identical images). PACGAN achieved an SSIM score of 0.602, which may initially seem lower when compared to previous works^36–48^ reporting values between 0.57 and 0.98. However, it is important to note that the obtained SSIM value is optimal in our specific case. Specifically, the optimal SSIM value between the real and synthetic images should be comparable to the score between pairs of real images (Real/Real in Table 1). A higher SSIM value would indicate that, on average, pairs of real and fake images are more similar than pairs of real images, hence not reflecting the real distribution. In this regard, our optimal SSIM score of 0.602 holds significance, as it closely aligns with the SSIM between pairs of real images (0.611), demonstrating a satisfactory level of similarity and realism for the synthetic brain MRI images generated by PACGAN, highlighting the model’s capability to generate realistic images. Furthermore, it is crucial, as suggested by Odena and colleagues^14^for the similarity between images from the synthetic distribution to be comparable to the similarity between images from the real distribution. A high SSIM value solely within the synthetic images would indicate that the generator of the GAN is afflicted with mode collapse, where it produces only a subset of images that are realistic enough to deceive the discriminator, failing to capture the full variability of the target distribution. In our case, the metrics computed using pairs of synthetic images (Fake/Fake in Table 1) are comparable to those computed between real images (Real/Real), indicating that PACGAN successfully avoids the problem of mode collapse. The model effectively captures the diversity and variability present in the target distribution, reinforcing its ability to generate diverse and realistic synthetic images.

To provide a benchmark for our results, we conducted an extensive literature review to identify previous studies that utilized GANs for brain MRI synthesis. Our analysis revealed that the majority of works focused on multi-modal synthesis^32,36–46,49^ to generate images representing different modalities. Conversely, only a few studies specifically aimed at data augmentation^47,48,50,51^. In terms of generating synthetic brain MRI of specific classes, we found only two studies^51,52^ that shared a similar objective to ours, targeting the generation of synthetic brain MRI for two classes: AD and HC. Notably, the majority of the other studies focused on generating images for a single class, rather than differentiating between classes. This comparison highlights the novelty of our study in generating synthetic brain MRI images for two distinct classes, and the limited number of existing studies that share this specific purpose. Han et al.^51^ employed a two-step approach to generate full-size brain MRI images of two classes: one progressive growing GAN was trained for each class (with and without tumors) separately. On the other hand, Islam et al.^52^ focused on generating synthetic brain positron emission tomography (PET) images representing three different stages: AD, mild cognitive impairment (MCI), and HC. They trained three GANs, each dedicated to generating images for one of the three classes, which tripled the computational time required. In comparison, our model addresses the task of data augmentation and has a distinct advantage. It only requires a single training process to generate images belonging to multiple classes. This approach enables PACGAN to learn a more comprehensive and faithful model of the real data distribution and the distinguishing features associated with each class. Consequently, our model offers increased efficiency and versatility in generating target images across a range of classes for data augmentation purposes.

To assess the practical utility of PACGAN-generated images, we conducted a downstream classification experiment designed to emulate a realistic low-data scenario, where access to large, labeled datasets is constrained due to privacy regulations or restrictive data sharing policies. Within this setting, we investigated whether synthetic images generated by PACGAN could serve as effective pretraining data to improve diagnostic accuracy in AD classification. Specifically, we compared two ResNet-18 classifiers: one pretrained on ImageNet, a standard benchmark for natural image features, and the other pretrained on 3000 synthetic brain MR images generated by PACGAN. Both models were subsequently fine-tuned on limited real data consisting of 100 and 200 T1-weighted MR images sampled from the National Alzheimer’s Coordinating Center (NACC) dataset. Importantly, the NACC dataset differs from the ADNI dataset (used to train PACGAN) in both diagnostic criteria and population characteristics, thereby introducing a domain shift that increases the complexity of the classification task. Despite this, across 100 bootstrap repetitions, the average AUC achieved by the classifiers pretrained on PACGAN-generated images was higher than that of those pretrained on ImageNet. Moreover, the CV across bootstrap repetitions was lower for PACGAN, indicating reduced variability in performance. These findings suggest that PACGAN-generated images may support more consistent classifier performance in low-data settings where real-domain pretraining data are unavailable.

We acknowledge several limitations in our study. Firstly, our demonstration of PACGAN focused on generating 2D images specific to a target class. However, considering that medical imaging data is typically acquired in 3D volumes, extending the PACGAN architecture to handle 3D data could provide a more comprehensive representation of the input data. This extension would enable PACGAN to compete with current state-of-the-art classifiers in the field, which often leverage 3D volumes for analysis and classification tasks. Moreover, the model has been trained on images from a restricted number of participants, thereby constraining its performance potential. Increasing the number of subjects in the training set could potentially enhance both the quality of the generated images and the classification performance of the discriminator. Finally, our evaluation of synthetic images involved the use of quantitative metrics and the assessment of classification performance by the discriminator, without the direct involvement of expert radiologists. However, it is important to note that the diagnostic accuracy of these synthetic images cannot be guaranteed. Therefore, further validation and assessment by medical professionals would be necessary to determine the clinical utility and diagnostic accuracy of the generated synthetic images.

In conclusion, PACGAN demonstrates its potential in two key aspects: (i) synthesizing brain MRI images of AD patients and HC, and (ii) accurately discriminating new instances between the two classes. The conditional synthesis capability of PACGAN offers the opportunity to generate a synthetic dataset that comes with native labels. Moreover, PACGAN exhibits versatility and applicability beyond brain MRI images. Indeed, its architecture can be adapted to generate high-resolution samples of various classes in the domain of natural and medical images. The flexibility of the model extends to accommodating images of resolutions other than the 256 $\:\times\:$ 256 size. The framework’s source code, available on the GitHub repository, is accompanied by Docker containerization, enabling researchers to readily adapt and deploy PACGAN for their specific image synthesis tasks.

## Methods

### Data source

PACGAN is a generalizable model that can be trained with different labeled datasets to generate and classify images belonging to two or more classes. For this study, we utilized data obtained from the Alzheimer’s Disease Neuroimaging Initiative (ADNI), which can be accessed through the LONI Image and Data Archive (IDA) research data repository. Access to the data, public and freely accessible, can be obtained by researchers through the website http://adni.loni.usc.edu/data-samples/adni-data/. Interested individuals are required to submit an online application form that contains the institutional affiliation of the investigator and the intended purpose of data usage. Diagnostic classification in ADNI is obtained based on the Mini-Mental State Examination (MMSE), Clinical Dementia Rating (CDR) score, and the Logical Memory II subscale of the Wechsler Memory Scale–Revised, with cutoff scores based on education^16^.

For this study, we included all follow-up data from all project phases (ADNI1, ADNI GO, ADNI2, ADNI3), and selected the T_1_-weighted MRI with a 3D acquisition type, selecting as *Research Group* “AD” and “CN”. We selected the data with image descriptions “MP-RAGE”, “MP-RAGE REPEAT”, “MP-RAGE SENSE2”, “Accelerated Sagittal MP-RAGE”, “Repeat Accelerated Sagittal MP-RAGE”, “MP-RAGE 24 FOV” and “MP-RAGE 24 FOV REPEAT”. This resulted in a total of 2727 downloaded 3D images (volumes). We carefully reviewed the dataset and discarded three duplicate volumes, eight corrupted volumes, and 232 volumes with various types of artifacts. Please refer to the Supplementary Methods for the corresponding Image Data IDs. To ensure comparability between the subject groups, we conducted further refinement through age-and-sex matching, as described in the *Training*,* validation and test set* section. This process led to the exclusion of additional 40 volumes. As a result, we obtained a final dataset consisting of 2444 volumes (679 AD and 1765 HC) to train, validate and test the model. These volumes were derived from a cohort of 521 subjects, comprising 234 males and 287 females, with ages ranging from 51 to 93 years (mean age 74.26).

To evaluate the utility of PACGAN, we used the National Alzheimer’s Coordinating Center (NACC) dataset^53^, which provides independently collected brain MR images under different clinical protocols. Diagnosis of AD in the NACC dataset follows different criteria than those used in ADNI and is defined by a combination of the cognitive status variable (NACCUDSD = 4) and the etiologic diagnosis variable (NACCALZD = 1), as outlined at https://www.naccdata.org/requesting-data/data-request-process. Access to NACC data is available to researchers upon request and acceptance of the data usage agreement, via https://naccdata.org/requesting-data/nacc-data.

To simulate a realistic low-data setting, we selected baseline MR images and randomly sampled 300 images from 300 unique subjects. This sample included 117 males and 183 females, aged 18 to 93 years (mean age 68.39). The selected images were divided into a test set of 100 images (28 AD, 72 CN) and a training set of 200 images (38 AD, 162 CN). To simulate a more constrained data setting, we further subsampled 100 images (20 AD, 80 CN) from the training set to create a smaller training set.

### MRI data preprocessing

We have co-registered each individual T_1_-weighted volume to the MNI152 standard template space at 1 mm voxel size using FSL^54^ (version 6.0). The co-registration process involved a linear registration with 9 degrees of freedom and trilinear interpolation using FSL’s FLIRT. The resulting co-registered T_1_-weighted volumes had a data matrix size of 182 $\:\times\:$ 218 $\:\times\:$ 182 and a voxel size of 1 mm $\:\times\:$ 1 mm $\:\times\:$ 1 mm. To achieve a cubic shape with a data matrix size of 256 $\:\times\:$ 256 $\:\times\:$ 256, each co-registered volume was processed using the Freesurfer tool mri_convert --conform. Additionally, the orientation of the volumes was changed from Left-Inferior-Anterior (LIA) to Right-Anterior-Superior (RAS) for improved visualization. Subsequently, we extracted the central axial slice corresponding to the slice number 127 from each for each co-registered T_1_-weighted volume. These axial slices were then concatenated into a single NIfTI volume with a size of 256 $\:\times\:$ 256 $\:\times\:$ #images, where #images represent the total number of images.

### PACGAN

The primary contribution of this study is the development and implementation of a proof-of-concept approach called Progressive ACGAN (PACGAN). This model combines the benefits of the multi-scale structure of Progressive Growing GAN with the ACGAN framework to synthesize full-size MR images (256 $\:\times\:$ 256) and enable the discriminator to classify each image according to its class.

### PACGAN architecture

**Fig. 2:**
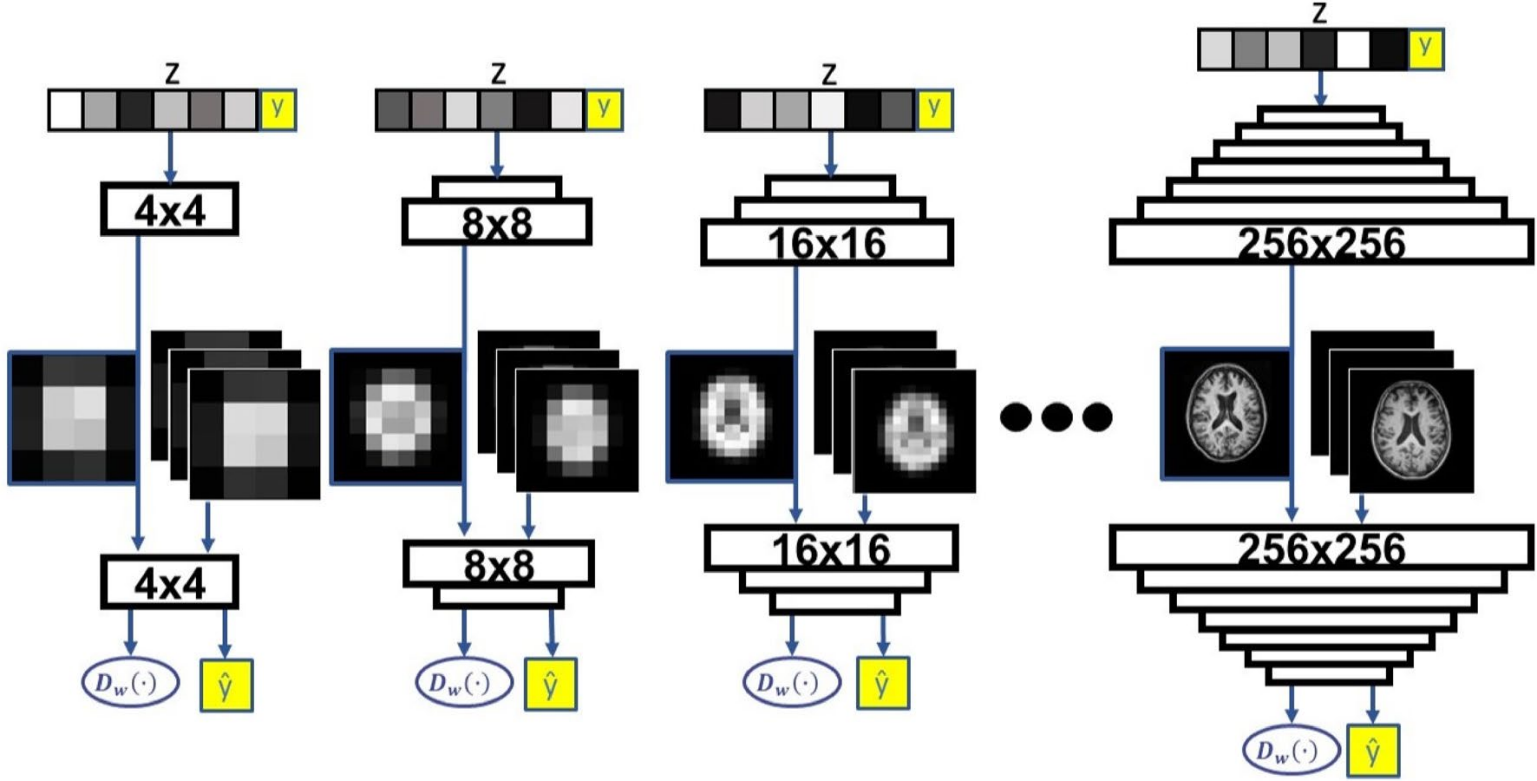
Architecture of the PACGAN model. The generator takes a random vector, denoted as (represented with reduced dimension for clarity), concatenated with the real label of the image to generate. In the algorithm, the label is embedded into a vector of elements () before being concatenated with. The discriminator produces two outputs: (i), the estimation of the input image’s realness, and (ii) the predicted class label.

The architecture, shown in Fig. 2, draws inspiration from the Progressive GAN proposed by Karras et al.^15^. The architecture of the two networks, schematized in Table 4 has been faithfully reproduced according to the original design. During the training, 3-layer blocks are progressively introduced to handle images of increasing resolution. To smoothly transition between layers, the output of the previous layer ($\:{O}_{prev}$) is either doubled (using nearest neighbor filtering) in the generator or halved (using average pooling) in the discriminator. To avoid sudden shock to the already trained layers, the outputs $\:{O}_{new}$ of the new layers are smoothly faded-in using a weighted sum $\:(1-\alpha\:)\cdot\:{O}_{prev}+\alpha\:\cdot\:{O}_{new}$, with $\:\alpha\:$ linearly increasing from 0 to 1. After each convolutional block (Conv 3 $\:\times\:$ 3) of the generator, the pixel-wise normalization of the feature vectors is performed.

To adapt the architecture for our specific purposes, two architectural modifications were made. Firstly, the network depth was adjusted. While the original Progressive Growing GAN was designed for 1024 $\:\times\:$ 1024 images, we modified it to work with 256 $\:\times\:$ 256 images by removing the last two blocks of the generator and the first two blocks of the discriminator. Additionally, the size of the feature maps within each block was suitably scaled. Note that our implementation can be easily modified to work with images of different sizes. Furthermore, the architectures were designed to accommodate both grayscale and RGB images, with a flexible number of channels $\:{n}_{ch}$.

The second modification involves the adaptation to the ACGAN architecture, enabling the model to handle datasets with a variable number of classes $\:{n}_{classes}$:

- In the *generator*, in addition to the latent vector $\:z$ of dimension $\:{z}_{dim}$, the input also included the label $\:y$ of the image to be generated. The label is embedded as a natural number in $\:em{b}_{dim}$ elements, resulting in the vector $\:cl{s}_{embedded}$ that is concatenated with $\:z$. For the sake of simplicity, in Fig. 2, the vector $\:cl{s}_{embedded}$ is denoted as $\:y$. The values of $\:{z}_{dim}$ and $\:em{b}_{dim}$ were determined through a grid-search process. For $\:{z}_{dim}$, we explored the values suggested by Goodfellow et al.^7^ ($\:{z}_{dim}=100$, for images of size 28 $\:\times\:$ 28 and 32 $\:\times\:$ 32) and by Karras et al.^15^ ($\:{z}_{dim}=512$, for images of size 1024 $\:\times\:$ 1024). Since our model is designed for 256 $\:\times\:$ 256 images, three values for $\:{z}_{dim}$ were investigated: 100, 300, and 512. To set the optimal value of $\:em{b}_{dim}$, we considered the natural numbers between 2 and 8. The optimal values were determined to be $\:{z}_{dim}=512$, and $\:em{b}_{dim}=3$, as discussed in the *Results* section.
- In the *discriminator* both real and fake images (normalized to values in the range [-1,1]) are provided as input. The discriminator produces two outputs:
  - $\:{D}_{w}(\cdot\:)$, which estimates the *realness* of the input image, following the architecture of the Progressive GAN. A fully connected layer with linear activation function is designed starting from the last 4 $\:\times\:$ 4 layers, as illustrated in Table 4.
  - $\:\widehat{y}$, which estimates the *class* of the input image through a deeper fully connected network (refer to Table 4). The softmax activation function is employed to handle multi-class classification problems with a general number of classes $\:{n}_{classes}$.

As recommended by Karras and colleagues^15^, a constant layer called Minibatch stddev is added at the end of the discriminator. This layer computes the average standard deviation of each feature over the minibatch, encouraging the minibatches of generated images to show similar statistical properties to the real ones. Additionally, we adhered to their suggestion of employing the Leaky Rectified Linear Activation (LReLU) with a leakiness of 0.2 in all layers of both the generator and discriminator networks, except for the last layers which utilize linear or softmax activation, as specified in Table 4.

### PACGAN implementation

The entire procedure is summarized in Algorithm 1, which was implemented in Python using the Pytorch framework. Following the scheme proposed by Goodfellow and colleagues^7^, each epoch consisted of alternating the optimization of *G* once with the optimization of *D* $\:{n}_{critic}$ times. Using $\:{n}_{critic}>1$ can yield a stronger discriminator, thereby challenging the generator to produce more realistic images. However, striking a balance is crucial, as an excessively proficient discriminator would impede constructive parameter updates in the generator, resulting in unsatisfactory training outcomes. Therefore, we set $\:{n}_{critic}=1$ for lower resolutions (4–64) and 2 for the higher resolutions (128,256). During the network growth process, the batch size was adjusted. A batch size of 32 was used for training at lower resolutions (4–16) and was reduced to 16 for the subsequent steps (resolution $\:\ge\:$32). For training, we employed the Adam optimizer^55^ with a learning rate of 0.001, $\:{\beta\:}_{1}=0.5$, $\:{\beta\:}_{2}=0.999$, while utilizing the GradScaler to prevent underflow.

Following the suggestion of Karras and colleagues^15^, we implemented the Wasserstein loss^56^ with gradient penalty^57^ (WGAN-GP) as the loss function. The Wasserstein distance can be implemented as $\:\frac{1}{m}\sum\:_{i=1}^{m}[{D}_{w}\left(G\right({z}^{\left(i\right)},{y}^{\left(i\right)}\left)\right)-{D}_{w}\left({x}^{\left(i\right)}\right)]$ under the assumption that the discriminator $\:{D}_{w}(\cdot\:)$ belongs to the parameterized family of K-Lipschitz functions. To enforce the Lipschitz constraint, the gradient penalty penalizes the discriminator whenever the gradient of $\:{D}_{w}(\cdot\:)$ exceeds a norm of 1. Due to the infeasibility of evaluating $\:{D}_{w}(\cdot\:)$ at all points in the input space, the gradient of $\:{D}_{w}\left(\widehat{x}\right)$ is computed, where $\:\hat{x} = \varepsilon x + (1 - \varepsilon )G(z,y)$ is a linear combination of real and generated data weighted by $\:\varepsilon$, randomly chosen between 0 and 1. In order to optimize the class estimation made by the discriminator, and encourage the generator to effectively utilize the input label $\:y$, the cross entropy (CE) between the real label and the estimated one was added to the loss function. As previously mentioned, in addition to $\:{D}_{w}(\cdot\:)$, which indicates the realness of the sample, the discriminator also outputs $\:\widehat{y}$, the estimated label. It should be noted that *D* receives as input both real samples and fake ones. Therefore, $\:{\widehat{y}}_{real}$ represents the estimated label of a real image, while $\:{\widehat{y}}_{fake}$ represents the estimation for a fake image.

In the discriminator loss, we assigned different weights to the gradient penalty and class loss to ensure their significance within the loss function. The gradient penalty was assigned a weight of $\:{\lambda\:}_{GP}=10$, consistent with the WGAN-GP^57^. To optimize class estimation, considering the different goals of the discriminator and the generator, we employed two different weights $\:{\lambda\:}_{CL{S}_{1}}$ and $\:{\lambda\:}_{CL{S}_{2}}$, with $\:{\lambda\:}_{CL{S}_{1}}=2\cdot\:{\lambda\:}_{CL{S}_{2}}$. For the discriminator, which aimed to correctly classify real images, optimizing the term $\:CE\left(y,{\widehat{y}}_{real}\right)$ was crucial and it was multiplied by $\:{\lambda\:}_{CL{S}_{1}}$, while the term $\:CE\left(y,{\widehat{y}}_{fake}\right)$ by $\:{\lambda\:}_{CL{S}_{2}}$. Conversely, for the generator, the primary objective was to generate images correctly classified by the discriminator. Hence, the weights were inverted with respect to the discriminator. The value of $\:{\lambda\:}_{CL{S}_{1}}$ was treated as a hyperparameter and determined through a grid search in combination with $\:{z}_{dim}$ and $\:em{b}_{dim}$. We explored values between 2 and 6, ultimately finding that $\:{\lambda\:}_{CL{S}_{1}}=4$ yielded the best results.

In addition to hyperparameter selection during the grid search, the validation set played a crucial role in determining the optimal number of training epochs for each step, to limit the problem of overfitting. During the validation process, we trained the model for 100 epochs per step, evaluating both the classification performance of the discriminator on new data (i.e., the validation set) and the ability of the generator to generate target images for each epoch. To determine the optimal number of epochs, we defined the $\:validation\_loss$ as shown at line 16 of Algorithm 1. This metric involved calculating the AUC between the real labels $\:y$ and the estimated labels for real and fake images ($\:{\widehat{y}}_{real}$ and $\:{\widehat{y}}_{fake}$, respectively) after completing 60% of the specified number of epochs for each step. Thus, at every epoch, the $\:validation\_loss$ was computed once 60 epochs of training were completed for each step. Upon completing each training phase, we identified the epoch with the highest $\:validation\_loss$ and designated it as the *best epoch*. The models obtained at the best epoch were saved and used as the starting point for training in the subsequent step (e.g., passing from training the 4 $\:\times\:$ 4 models to 8 $\:\times\:$ 8 models, as indicated in line 3 of Algorithm 1). Therefore, the validation set served two purposes: (i) the selection of hyperparameters during the grid search, schematized in Supplementary Tables 1, and (ii) determining the optimal number of training epochs for each phase, to obtain a model with enhanced generalization on new data.

### Training, validation, and test set

To ensure balance and comparability, patients and controls were matched within each group based on age and sex. The age matching between AD and HC was assessed using the Mann-Whitney U test, while the $\:{\chi\:}^{2}$ p-value was employed for the sex matching. Initially, matching was assessed for the entire population by systematically removing one male and one female for AD patients and 2 females for HC subjects within predefined age ranges ($\:49-60$ years for AD, $\:73-83$ years for HC) until all p-values were not significant ($\:\ge\:0.05$). The ranges were defined based on the differences observed between the two distributions. This phase resulted in the removal of 40 images (6 AD and 34 HC), obtaining a p-value of 0.1322 for the Mann-Whitney U test, and 0.0524 for the $\:{\chi\:}^{2}$ test.

**Table 4:** Architecture details of the generator and discriminator in the PACGAN framework for generating 256 × 256 images. The generator takes the random vector z and the embedded label of the image to be generated, $\:cl{s}_{embedded}$ as input. It generates an image of size 256 × 256 with a variable number of channels, denoted as Nch. On the other hand, the discriminator receives images of size n_ch_ × 256 × 256 and produces two outputs: the realness of the input image, denoted as $\:{D}_{w}\left(\cdot\:\right)$, and the relative class estimation, denoted as $\:\widehat{y}$. Both networks consist of convolutional (Conv) and fully connected (FC) layers, with activation functions (Act.) such as leaky rectified linear activation function (LReLU), linear, or softmax.

Generator	Act.	Output shape	Params	Discriminator	Act.	Output shape	Params
z | $\:cl{s}_{embedded}$	$\:-$	522 $\:\times\:$ 1 $\:\times\:$ 1	$\:-$	Input image	$\:-$	$\:{n}_{ch}$ $\:\times\:$ 256 $\:\times\:$ 256	$\:-$
Conv 4 $\:\times\:$ 4	LReLU	512 $\:\times\:$ 4 $\:\times\:$4	4.28 M	Conv 1 $\:\times\:$ 1	LReLU	16 $\:\times\:$ 256 $\:\times\:$ 256	1k
Conv 3 $\:\times\:$ 3	LReLU	512 $\:\times\:$ 4 $\:\times\:$ 4	2.36 M	Conv 3 $\:\times\:$ 3	LReLU	16 $\:\times\:$ 256 $\:\times\:$ 256	4.6k
Upsample	$\:-$	512 $\:\times\:$ 8 $\:\times\:$ 8	$\:-$	Conv 3 $\:\times\:$ 3	LReLU	32 $\:\times\:$ 256 $\:\times\:$ 256	9.2k
Conv 3 $\:\times\:$ 3	LReLU	512 $\:\times\:$ 8 $\:\times\:$ 8	2.36 M	Downsample	$\:-$	32 $\:\times\:$ 128 $\:\times\:$ 128	$\:-$
Conv 3 $\:\times\:$ 3	LReLU	512 $\:\times\:$ 8 $\:\times\:$ 8	2.36 M	Conv 3 $\:\times\:$ 3	LReLU	32 $\:\times\:$ 128 $\:\times\:$ 128	18.4k
Upsample	$\:-$	512 $\:\times\:$ 16 $\:\times\:$ 16	$\:-$	Conv 3 $\:\times\:$ 3	LReLU	64 $\:\times\:$ 128 $\:\times\:$ 128	37k
Conv 3 $\:\times\:$ 3	LReLU	256 $\:\times\:$ 16 $\:\times\:$ 16	1.18 M	Downsample	$\:-$	32 $\:\times\:$ 64 $\:\times\:$ 64	$\:-$
Conv 3 $\:\times\:$ 3	LReLU	256 $\:\times\:$ 16 $\:\times\:$ 16	590k	Conv 3 $\:\times\:$ 3	LReLU	64 $\:\times\:$ 64 $\:\times\:$ 64	74k
Upsample	$\:-$	256 $\:\times\:$ 32 $\:\times\:$ 32	$\:-$	Conv 3 $\:\times\:$ 3	LReLU	128 $\:\times\:$ 64 $\:\times\:$ 64	147k
Conv 3 $\:\times\:$ 3	LReLU	128 $\:\times\:$ 32 $\:\times\:$ 32	295k	Downsample	$\:-$	128 $\:\times\:$ 32 $\:\times\:$ 32	$\:-$
Conv 3 $\:\times\:$ 3	LReLU	128 $\:\times\:$ 32 $\:\times\:$ 32	147k	Conv 3 $\:\times\:$ 3	LReLU	128 $\:\times\:$ 32 $\:\times\:$ 32	295k
Upsample	$\:-$	128 $\:\times\:$ 64 $\:\times\:$ 64	$\:-$	Conv 3 $\:\times\:$ 3	LReLU	256 $\:\times\:$ 32 $\:\times\:$ 32	590k
Conv 3 $\:\times\:$ 3	LReLU	64 $\:\times\:$ 64 $\:\times\:$ 64	74k	Downsample	$\:-$	256 $\:\times\:$ 16 $\:\times\:$ 16	$\:-$
Conv 3 $\:\times\:$ 3	LReLU	64 $\:\times\:$ 64 $\:\times\:$ 64	37k	Conv 3 $\:\times\:$ 3	LReLU	256 $\:\times\:$ 16 $\:\times\:$ 16	1.18 M
Upsample	$\:-$	64 $\:\times\:$ 128 $\:\times\:$ 128	$\:-$	Conv 3 $\:\times\:$ 3	LReLU	512 $\:\times\:$ 16 $\:\times\:$ 16	2.36 M
Conv 3 $\:\times\:$ 3	LReLU	32 $\:\times\:$ 128 $\:\times\:$ 128	18.4k	Downsample	$\:-$	512 $\:\times\:$ 8 $\:\times\:$ 8	$\:-$
Conv 3 $\:\times\:$ 3	LReLU	32 $\:\times\:$ 128 $\:\times\:$ 128	9.2k	Conv 3 $\:\times\:$ 3	LReLU	512 $\:\times\:$ 8 $\:\times\:$ 8	2.36 M
Upsample	$\:-$	32 $\:\times\:$ 256 $\:\times\:$ 256	$\:-$	Conv 3 $\:\times\:$ 3	LReLU	512 $\:\times\:$ 8 $\:\times\:$ 8	2.36 M
Conv 3 $\:\times\:$ 3	LReLU	16 $\:\times\:$ 256 $\:\times\:$ 256	4.6k	Downsample	$\:-$	512 $\:\times\:$ 4 $\:\times\:$ 4	$\:-$
Conv 3 $\:\times\:$ 3	LReLU	16 $\:\times\:$ 256 $\:\times\:$ 256	2.3k	Minibatch stddev	$\:-$	513 $\:\times\:$ 4 $\:\times\:$ 4	$\:-$
Conv 1 $\:\times\:$ 1	linear	$\:{n}_{ch}$ $\:\times\:$ 256 $\:\times\:$ 256	1.5k	Conv 3 $\:\times\:$ 3	LReLU	512 $\:\times\:$ 4 $\:\times\:$ 4	2.36 M
Tot params			13.7 M	Conv 4 $\:\times\:$ 4	LReLU	512 $\:\times\:$ 1 $\:\times\:$ 1	4.2 M
				FC $\:\left({D}_{w}\left(\cdot\:\right)\right)$	linear	1 $\:\times\:$ 1 $\:\times\:$ 1	513
				FC	linear	150 $\:\times\:$ 1 $\:\times\:$ 1	76.9k
				FC $\:\left(\widehat{y}\right)$	Softmax	$\:{n}_{classes}$ $\:\times\:$ 1 $\:\times\:$ 1	151
				Tot params			16.1 M

**Algorithm 1:**
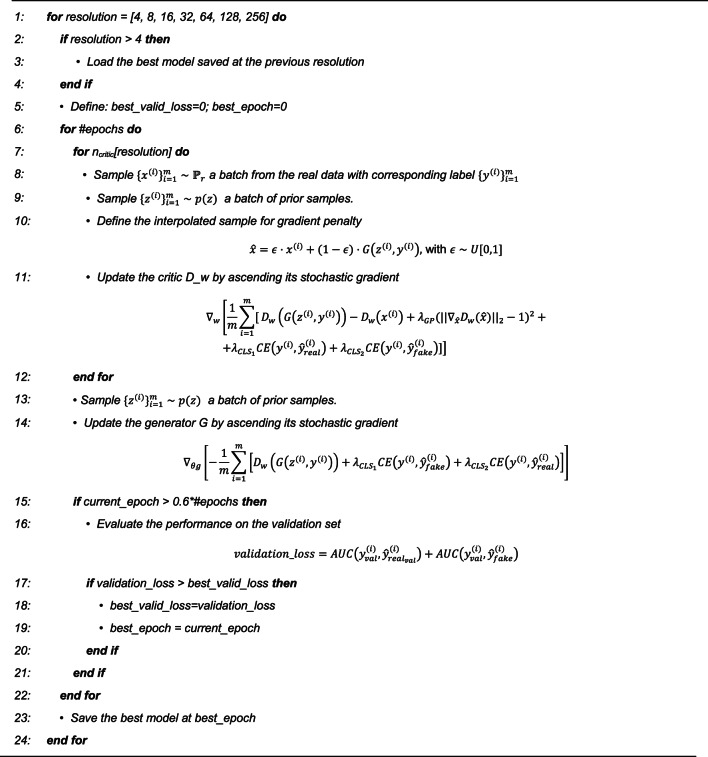
PACGAN training procedure. The training process consists of multiple phases, during which the algorithm operates on images of increasing resolution, ranging from 4 × 4 to 256 × 256. For each resolution, training is performed for a fixed number of epochs, denoted as #epochs. During each epoch, the discriminator is trained n_critic_ times (with n_critic_ varying depending on the resolution), while the generator is trained once. After completing 60% of the total training epochs, the validation_loss is computed for each epoch to identify the best_epoch, which corresponds to the model at the current resolution that exhibits superior generalization performance on the validation set. The model obtained at the best_epoch is considered the best model and serves as the starting point for training the subsequent resolution.

Subsequently, the matched subjects were divided into training, validation, and test set. To avoid data leakage^58,59, ^which can introduce bias and compromise model evaluation, we took precautions to ensure that images from the same subject were grouped together (i.e., in the training, validation, or test set). This was achieved through the use of a stratified holdout approach, exploiting the StratifiedGroupKFold function in scikit-learn^60^. The division of images was carried out in two sequential steps. In the first step, we employed a 5-fold stratified cross-validation scheme grouped by subjects, whereby 20% of the images were assigned to the test set, while the remaining 80% were allocated for training and validation. This first step was iteratively repeated until age and sex matching reached non-significance, as indicated by p-values from the Mann-Whitney U test for age and the $\:{\chi\:}^{2}$ test for sex. In the second step, the 80% subset of images was further split into the training and the validation set. Within this already matched set, we applied the aforementioned stratified holdout procedure, keeping 20% of the images as the validation set, ensuring that images of the same subject remained in the same set.

The Image Data IDs of the images used as training, validation, and test sets are listed in the Supplementary Methods.

### Assessment metrics

#### Quality of synthetic images

The quality evaluation of the generated images encompasses both visual and quantitative assessment. Considering that there is not a GAN evaluation measure better than the others^29^, we implemented multiple scores that measure semantic similarity: FID, KID, and SSIM.

The FID, which has been shown to be consistent with human judgments^29^, quantifies the similarity between feature vectors extracted from images belonging to two distributions. To compute FID, a set of generated samples and real images are embedded into a feature space using a specific layer of Inception Net^61^. The mean and covariance of the embedding for fake and real data are computed by treating the embedding layer as a continuous multivariate Gaussian distribution. The Fréchet distance, also known as Wasserstein-2 distance, is calculated between these two Gaussian distributions, defined as:

$$\:FID(r,g)=\parallel\:{\mu\:}_{r}-{\mu\:}_{g}{\parallel\:}_{2}^{2}+Tr\left({\sigma\:}_{r}+{\sigma\:}_{g}-2({\sigma\:}_{r}{\sigma\:}_{g}{)}^{\frac{1}{2}}\right),$$

where Tr represents the trace of the matrix, $\:({\mu\:}_{r},{\sigma\:}_{r})$ and $\:({\mu\:}_{g},{\sigma\:}_{g})$ denote the mean and covariance of the real $\:\left(r\right)$ and generated $\:\left(g\right)$ distributions, respectively. A lower FID score indicates a smaller distance between the synthetic and real data distributions. We exploited the *TorchMetrics*^62^ implementation of FID, which takes as input the two image distributions of interest, saturating to zero negative values. It is important to note that when the number of images is limited, the FID metric may be biased, and meaningful comparisons can only be made if it is computed with the same number of samples^19^.

The KID serves as an unbiased alternative to FID. Similar to FID, it employs the Inception Net to extract feature vectors from real and fake images. However, KID computes the distance between these feature vectors using a polynomial kernel:

$$\:K\left(\varphi\:\right(x),\varphi\:(y\left)\right)={\left(\frac{1}{d}\varphi\:\left(x{)}^{T}\varphi\:\right(y)+1\right)}^{3},$$

where $\:\varphi\:\left(x\right)$ and $\:\varphi\:\left(y\right)$ represent the Inception representations of real ($\:x$) and fake ($\:y$) images, and $\:d$ is the dimension of the representation. We utilized the *TorchMetrics* implementation of KID, which takes as input the two image distributions and divides them into multiple batches to compute the score several times, estimating the mean and standard deviation. Negative values have been saturated to zero. The KID computation was performed twice: once with the entire distribution of generated data and once with images of the same class, using a batch size of 50. It should be noted that the same approach cannot be applied to FID, as reducing the number of images involved in the computation would inflate the score, leading to inaccurate results. Both FID and KID metrics demand images of size 299 $\:\times\:$ 299 RGB to align with Inception Net’s input specifications. Consequently, we resized and converted the images of interest to RGB format by replicating grayscale images in three channels.

The SSIM is a perceptual similarity measure widely used to evaluate the realness of the synthetic brain MRI images^35, ^that seeks to exclude features of a picture that are not crucial for human perception^20^. It compares corresponding pixels and their neighborhoods in two images, denoted as $\:x$ and $\:y$, using three quantities: luminance (I), contrast (C), and structure (S):

$$\:\begin{array}{c}I(x,y)=\frac{2{\mu\:}_{x}{\mu\:}_{y}+{C}_{1}}{{\mu\:}_{x}^{2}+{\mu\:}_{y}^{2}+{C}_{1}}C(x,y)=\frac{2{\sigma\:}_{x}{\sigma\:}_{y}+{C}_{2}}{{\sigma\:}_{x}^{2}+{\sigma\:}_{y}^{2}+{C}_{2}}S(x,y)=\frac{{\sigma\:}_{xy}+{C}_{3}}{{\sigma\:}_{x}{\sigma\:}_{y}+{C}_{3}}\end{array}$$

Here, $\:{\mu\:}_{x}$, $\:{\mu\:}_{y}$, $\:{\sigma\:}_{x}$ and $\:{\sigma\:}_{y}$ represent the mean and standard deviations of pixel intensities within a local image patch centered at either $\:x$ or $\:y$ (typically a square neighborhood of 5 pixels). $\:{\sigma\:}_{xy}$ denotes the sample correlation coefficient between corresponding pixels in the patches centered at $\:x$ and $\:y$. The constants $\:{C}_{1}$, $\:{C}_{2}$, and $\:{C}_{3}$ are small values added for numerical stability. The SSIM score is obtained by combining the three quantities:

$$\:SSIM(x,y)=I(x,y{)}^{\alpha\:}C(x,y{)}^{\beta\:}S(x,y{)}^{\gamma\:}$$

Typically, $\:\alpha\:$, $\:\beta\:$, and $\:\gamma\:$ are set to 1. The resulting SSIM index ranges from 0 to 1, where 1 indicates that the two images are identical. Computation of the SSIM score was performed using the *scikit-image*^60^ implementation, which requires a pair of images as input. To compute the mean and standard deviation, the computation was repeated for 1000 sample pairs, following the methodology described by Odena and colleagues^14^.

### Classification metrics

Although the primary objective of the generator is to synthesize realistic images, in the context of PACGAN (and conditional GANs in general) it is also crucial to learn to represent images differently based on their labels. In our case, this means that synthetic brain MRIs of healthy subjects should exhibit discernible differences from those of AD patients, reflecting the actual differences between the two groups. To assess this ability, we used the AUC.

The AUC serves as a summary of the classification performance and is commonly used to compare different classifiers. For binary classification problems, the confusion matrix is constructed, defining the number of True Positive (TP — positive class data point correctly classified as positive), True Negative (TN — negative class data point correctly classified as negative), False Positive (FP — negative class data point incorrectly classified as positive) and False Negative (FN — positive class data point incorrectly classified as negative). The ROC curve is a graphical representation that depicts the True Positive Rate $\:\left(TPR=\frac{TP}{TP+FN}\right)$ and the False Positive Rate $\:\left(FPR=\frac{FP}{FP+TN}\right)$ at various classification thresholds. The AUC is the area under the ROC curve and was computed using the roc_auc_score function provided by scikit-learn. A higher AUC value, closer to 1, indicates better predictive performance of the discriminator.

To evaluate the performance of the discriminator, we also trained multiple ResNet models on the same training set used to train PACGAN and assessed their performance on the same test set. Specifically, we fine-tuned ResNet-18, ResNet-34, ResNet-50, and ResNet-101 for 10 epochs using the Adam optimizer with a learning rate of 0.001.

### Utility assessment

To assess the practical utility of PACGAN in a downstream classification task, we investigated whether pretraining a classifier on synthetic brain MR images generated by PACGAN could improve performance in the diagnosis of Alzheimer’s disease. We focused on a realistic scenario commonly encountered in the medical imaging domain, where researchers need to train classification models using only limited amounts of labeled data. In such cases, pretraining on external datasets is a well-established strategy to enhance model performance. However, when pretraining relies on large datasets of natural (non-medical) images — such as ImageNet — the learned representations may not transfer effectively to medical tasks due to significant domain mismatch. Although large, domain-relevant medical datasets could provide more suitable pretraining data, they are frequently inaccessible due to privacy concerns or data sharing limitations, and may not correspond directly to the specific task at hand.

**Fig. 3:**
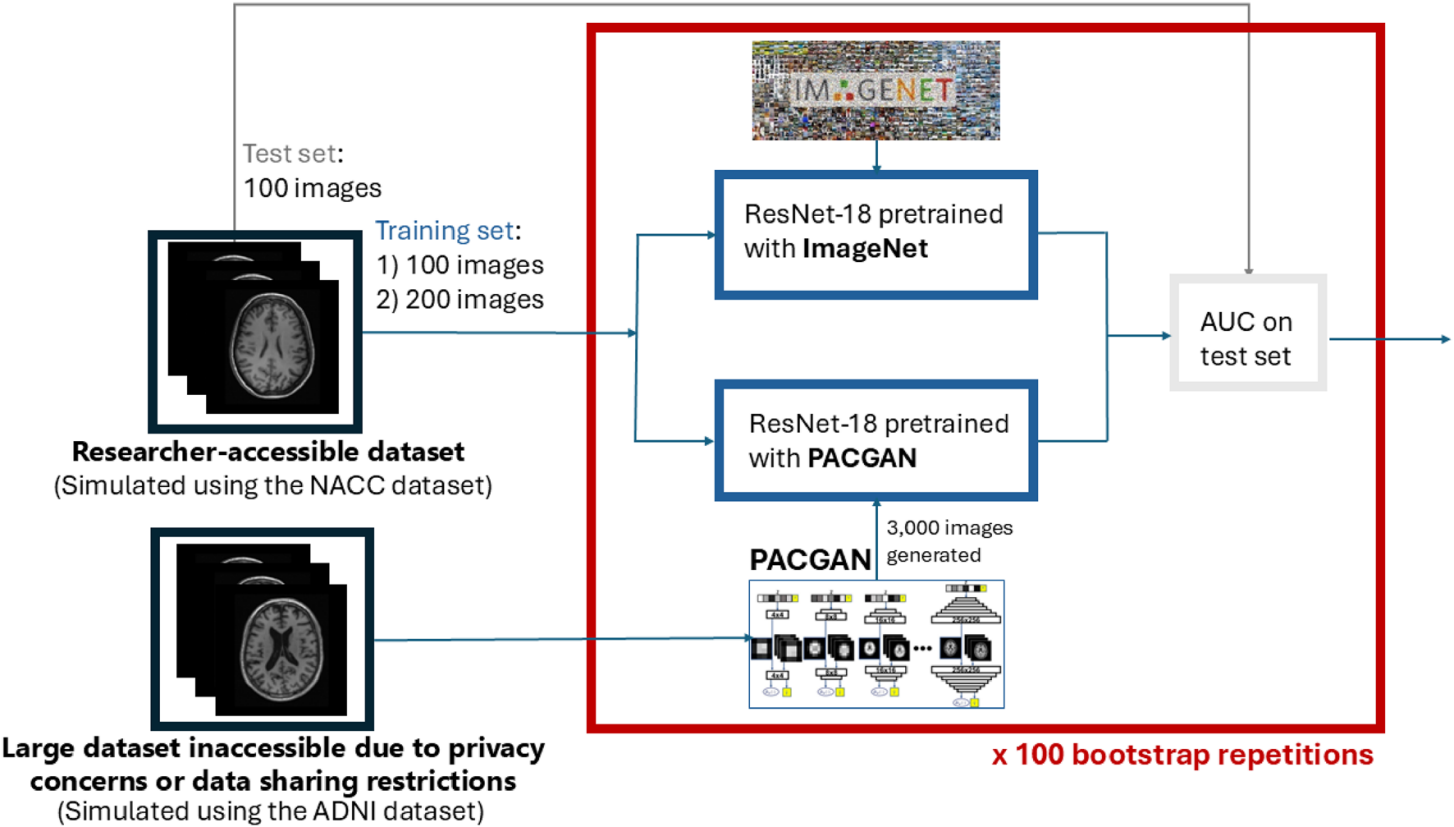
Experimental scheme to assess the utility of the synthetic images generated by PACGAN for the pretraining of a ResNet-18 classifier. The training was performed with two training sets, repeated for 100 bootstrap repetitions, and tested on a test set using the area under the ROC curve (AUC).

To simulate this scenario, we used the NACC dataset and applied the same preprocessing pipeline as with the ADNI dataset. From the preprocessed data, we constructed two training subsets consisting of 100 and 200 MR images. Given the limited training data, we selected ResNet-18 as the classifier architecture due to its relatively shallow depth, which helps mitigate overfitting in low-data regimes. We compared two pretraining strategies for ResNet-18: (i) Pretraining on ImageNet, exploiting the model provided by torchvision.models, and (ii) pretraining on 3000 synthetic MR images for 100 epochs, with early stopping using a patience of 20 epochs. Each pretrained model was fine-tuned for 10 epochs on the two NACC training sets (100 and 200 images) and evaluated on the test set. To assess sample variability, we repeated this process applying 100 bootstrap iterations. Specifically, for each training set size, we generated 100 resampled training subsets, allowing duplicates, to account for fluctuations due to sampling. For each iteration, we computed the AUC. After all iterations, we calculated the CV to quantify the relative variability in performance. An overview of the experimental setup is shown in Fig. 3.

### PACGAN hyperparameter tuning

To determine the optimal values for the three hyperparameters of the PACGAN model ($\:{z}_{dim}\in\:\{100,300,512\}$, $\:em{b}_{dim}\in\:\{2,3,4,5,6,7,8\}$, $\:{\lambda\:}_{cls}\in\:\{2,3,4,5,6\}$), we employed the grid search method. This involved conducting 105 training configurations on an NVIDIA A100 Tensor Core GPU, resulting in a total computational time of 630 h. For each training configuration, we evaluated the AUC on the validation set and measured FID, KID, and SSIM to assess image similarity. These metrics were computed both between images from the two distributions (synthetic and real ones) and between images from the same distribution.

The selection of the best model was based on the classification performance of the discriminator, assessed through the AUC, and the quality of the synthetic images. Considering the importance of accurately capturing the structural differences between the two classes during the generation of synthetic data and that the discriminator serves as a guide for the generation process, we prioritized its classification performance when selecting the final model. For this reason, we chose the hyperparameter configuration that reached the highest AUC score on the validation set as the best configuration.

Subsequently, the resulting model was re-trained on the combined training and validation set. The optimal number of epochs determined during the validation phase (as outlined in Algorithm 1) was used for this re-training process. Finally, the performance of the model was evaluated on the test set.

## Supplementary Information

Below is the link to the electronic supplementary material.

Supplementary Material 1

## Data Availability

The dataset used to train PACGAN and to assess the quality of its synthetic images is available in the Alzheimer’s Disease Neuroimaging Initiative (ADNI) repository, https://adni.loni.usc.edu/data-samples/adni-data. The synthetic images generated and analysed during the current study are available in 10.5281/zenodo.8276786. The dataset used to assess the utility of PACGAN is available through the National Alzheimer’s Coordinating Center (NACC) site at https://naccdata.org/requesting-data/nacc-data. The ResNet-18 model pretrained on 3000 PACGAN-generated images is available at: 10.5281/zenodo.15614653.
