# Supplementary material for: High-resolution conditional MR image synthesis through the PACGAN framework
Source: Sci Rep. 2025 Oct 1;15:34241. doi: 10.1038/s41598-025-16257-1 (PMC12489083; doi:10.1038/s41598-025-16257-1)
Supplement: Supplementary file 1 — Supplementary Material 1 [file 41598_2025_16257_MOESM1_ESM.pdf]

Supplement to:

High-resolution conditional MR image synthesis through the PACGAN framework

Authors: Matteo Lai, Chiara Marzi, Luca Citi, and Stefano Diciotti

Supplementary Methods

The dataset used to train the PACGAN was composed of 2444 images (belonging to 521 subjects), while a total of 2727 MR volumes were downloaded from the Alzheimer's Disease Neuroimaging Initiative (ADNI) database (adni.loni.usc.edu). Here is the list of the *Image Data ID*s that were discarded:

- 3 images were doubled
  I58423, I70013, I41449;

- 8 images were corrupted:
  I424987, I142384, I47811, I135611, I59843, I191337, I379681, I35210;

- 232 images had artifacts of various types:
  I139971, I11888, I130081, I71225, I71226, I19772, I1227908, I42743, I1152416, I166224, I103999, I47591, I27223, I103962, I20168, I1034852, I191674, I104322, I69143, I73617, I1440206, I167081, I97654, I41044, I11567, I42727, I1152419, I1037249, I84667, I17813, I116518, I16598, I335995, I1005122, I20762, I97653, I120216, I280361, I173505, I32531, I55658, I70391, I18220, I196664, I352726, I60125, I55900, I226354, I58145, I1334456, I98715, I97652, I1037252, I116517, I48331, I35221, I139970, I37351, I84666, I40915, I119758, I133523, I29363, I346800, I31238, I39503, I64129, I133026, I11887, I372468, I159297, I137194, I298718, I104263, I70290, I69144, I24264, I138225, I15861, I119843, I119756, I25983, I44148, I229358, I32054, I19990, I57658, I92792, I1488791, I812645, I130082, I206944, I15497, I20169, I1004740, I1004681, I137193, I415211, I54594, I53718, I25984, I226346, I1175747, I1334455, I662487, I24263, I29229, I48224, I133029, I23573, I88591, I20682, I36797, I152888, I137192, I168302, I167080, I109471, I1278640, I104000, I371971, I119842, I207257, I90193, I17619, I266030, I311332, I207263, I1047619, I1440205, I32177, I92791, I86851, I1018889, I12465, I58146, I88590, I35355, I358820, I135850, I963558, I29230, I15498, I129094, I120215, I1117156, I42728, I117847, I40916, I70289, I373513, I35220, I19659, I1266356, I54261, I72991, I20761, I15860, I74883, I66499, I206941, I142726, I15959, I104321, I1027631, I30221, I91584, I371710, I33899, I18219, I17795, I103961, I133028, I57178, I44828, I922723, I59845, I55657, I128220, I82873, I1027315, I28543, I1191372, I63979, I131841, I119757, I36798, I42742, I1075536, I37350, I70390, I72992, I106049, I48225, I82872, I165859, I19771, I106050, I103963, I32530, I137190, I266032, I14145, I133522, I100003, I150903, I375334, I73616, I39502, I152902, I24734, I16392, I234764, I57662, I56100, I107542, I229357, I914999, I74882, I1037250, I108784, I98714, I55659, I47592, I117848, I152901, I371711, I14146, I896821, I37352, I305993, I103575;

- 40 images were removed to obtain an age-and-sex matched dataset:
  I1485104, I968225, I81676, I166845, I369303, I71878, I42804, I118225, I1005735, I27222, I287488, I1284408, I1400194, I160489, I228875, I1463003, I38955, I139457, I895056, I114885, I225441, I138316, I176996, I142878, I51790, I30848, I126004, I33158, I495940, I1478784, I114996, I1060804, I18896, I14816, I136486, I361330, I1159023, I142515, I142725, I885655;

Among the 2444 images, 1955 were used to train and validate the model, and 489 were kept to test the models, as detailed in the *Training, validation, and test set* section.

Training and validation set

The *Image Data IDs* of the 1955 images used as training and validation set are listed below:

I13722, I28561, I55275, I114210, I144446, I144447, I173732, I13721, I28560, I55276, I114209, I173739, I238628, I303067, I863056, I1221051, I14437, I29705, I55771, I114997, I143221, I173460, I14438, I29706, I114996, I143220, I173461, I240808, I304792, I371997, I423203, I569631, I15948, I32909, I60124, I116575, I147113, I15949, I32910, I116574, I147114, I33171, I116119, I16391, I16390, I33170, I116120, I18211, I37161, I64466, I116764, I149924, I185090, I18210, I37162, I64465, I116763, I149923, I185093, I243903, I322440, I23591, I49010, I88804, I23590, I49011, I88805, I25952, I49023, I78379, I124843, I25953, I49024, I78380, I124844, I26170, I54260, I26171, I30875, I60268, I84436, I130789, I30874, I60267, I84437, I130790, I829296, I1140407, I40083, I70096, I99432, I166431, I40082, I70097, I99431, I136592, I136591, I166429, I233432, I287488, I361330, I418023, I491258, I888008, I1236679, I254579, I273216, I290926, I391153, I573756, I1075136, I257274, I274577, I297354, I340044, I395109, I259658, I274826, I299157, I346114, I397605, I573592, I788894, I260579, I277129, I301753, I398526, I575601, I346236, I358612, I372814, I398678, I835740, I988538, I1270100, I840235, I1167388, I861965, I1229529, I892212, I1255474, I1017836, I1151456, I1249336, I1514449, I1226294, I1154566, I1430377, I863251, I1178908, I1255144, I965015, I973278, I974338, I977365, I1001084, I1185102, I1227039, I1012896, I1205679, I1043769, I1083042, I1254307, I1427086, I1456305, I1483860, I1495816, I11645, I26116, I45004, I98933, I143295, I11646, I26115, I45003, I98932, I143296, I16992, I35240, I36118, I57021, I64128, I112818, I116549, I155040, I155059, I196443, I266980, I16991, I36117, I112817, I116548, I155038, I155058, I196444, I266979, I47507, I77589, I83444, I118313, I23572, I47508, I77590, I83445, I118314, I43188, I76567, I102672, I140378, I43189, I76568, I102671, I140379, I17505, I25790, I44485, I75182, I119098, I207800, I266902, I337725, I25791, I44486, I75183, I119099, I207804, I266907, I337717, I561269, I17794, I25816, I25865, I25817, I25866, I17867, I31252, I17868, I30064, I23468, I44455, I71755, I118483, I217897, I217892, I267549, I336755, I23469, I71754, I118482, I267546, I336753, I395974, I552101, I249408, I265126, I289849, I336210, I389364, I542294, I248518, I264984, I293812, I335304, I390350, I268916, I286457, I311254, I349322, I405707, I574352, I279473, I298511, I319635, I362890, I281885, I303084, I326302, I361293, I416632, I639536, I287990, I310928, I336553, I322002, I346361, I362016, I394331, I12616, I28050, I53700, I106387, I12617, I28051, I53701, I106388, I36411, I37761, I69912, I69921, I91605, I91614, I135827, I135849, I166158, I36412, I37762, I69913, I69922, I91606, I91615, I135828, I166157, I955206, I1291745, I37270, I38482, I71869, I71878, I91623, I91625, I91633, I136457, I136486, I166407, I664578, I37271, I38483, I71870, I71880, I91624, I136458, I136487, I166406, I664580, I42344, I77664, I103430, I144062, I42345, I77665, I103431, I144063, I925944, I1064236, I1244513, I902659, I1060837, I1428189, I942907, I1084935, I916119, I1060804, I1244529, I937847, I1081546, I983493, I1327191, I986872, I1342528, I1011962, I1344288, I154019, I154018, I156154, I156155, I205837, I156156, I205835, I157179, I157180, I283910, I319209, I957745, I915862, I940882, I1132797, I1281631, I1501111, I898881, I985197, I1160021, I998447, I1170118, I1009810, I238538, I253521, I308380, I274419, I642412, I290409, I312873, I336710, I365237, I418174, I665222, I296876, I914397, I1166752, I1225000, I297694, I313947, I368891, I377214, I421361, I784921, I296784, I314138, I375165, I990113, I296864, I340740, I368949, I369617, I308179, I360331, I373412, I390455, I1189178, I1221673, I12327, I27395, I49507, I104827, I12328, I27394, I49506, I104828, I13004, I27049, I13003, I27048, I12963, I12962, I15323, I32171, I55142, I111593, I145394, I191657, I371705, I15324, I32172, I55143, I111594, I145393, I191659, I371702, I16024, I32938, I56836, I111028, I149716, I188697, I16023, I32937, I56835, I111029, I149715, I188695, I17400, I36344, I60877, I112894, I148261, I17401, I36345, I60876, I112895, I148260, I191339, I31576, I56821, I88876, I32029, I56822, I88877, I907767, I1224468, I899473, I1072377, I907712, I1072841, I1254369, I948081, I1152910, I1299912, I969431, I1002374, I1037394, I1491025, I1039134, I1346790, I1037417, I1254386, I1440034, I1481579, I820302, I1173060, I820315, I1172863, I1000691, I1359936, I1194377, I1194293, I1359954, I1261605, I1256802, I1263249, I1426627, I1285276, I1435744, I1446304, I1458511, I262075, I296616, I320521, I266626, I296884, I315801, I348162, I273497, I296640, I316621, I353124, I408994, I580979, I301391,

I321526, I343575, I422891, I306067, I327944, I348651, I382180, I258946, I272543, I300353, I341970, I396531, I269274, I288739, I306123, I347146, I401664, I569607, I280784, I297110, I322353, I359781, I412890, I300337, I317432, I343284, I363191, I343920, I358058, I372597, I400430, I345558, I345557, I958011, I966268, I1332407, I983428, I996377, I1029584, I1224869, I1329968, I1045619, I10590, I24016, I42623, I95362, I139123, I167931, I10591, I24018, I24017, I42622, I42624, I95361, I139122, I167930, I167928, I12466, I27210, I51110, I104454, I142667, I173062, I27209, I51112, I51111, I104453, I104452, I142666, I173061, I17633, I17632, I37449, I60828, I17631, I37448, I37447, I60827, I17668, I38973, I64485, I116498, I17669, I38974, I64486, I64484, I116499, I44827, I27056, I58191, I85022, I132051, I161155, I207546, I27057, I58190, I85023, I132052, I161154, I207551, I844181, I1184047, I895056, I1142366, I1264767, I1485104, I1223029, I1342083, I1484349, I1236085, I987370, I1158135, I929044, I1135165, I1251421, I942773, I1263811, I974673, I996485, I1002815, I1003993, I1186737, I1004030, I1032282, I1195981, I27607, I53518, I78349, I124649, I159930, I27608, I53519, I78348, I124650, I28111, I54958, I81676, I126423, I160489, I215918, I28110, I54957, I81675, I126424, I160488, I215913, I35190, I58968, I87447, I133459, I35191, I58969, I87448, I133460, I885655, I1195083, I1479560, I828694, I1146857, I1464695, I958727, I1287192, I9487, I18233, I34644, I86850, I162251, I209535, I9488, I18234, I34643, I162250, I209543, I11370, I135609, I166285, I215697, I11371, I41045, I91583, I135610, I166286, I215698, I10933, I20659, I40995, I93249, I137015, I166618, I216139, I10934, I20658, I40994, I93248, I137014, I166617, I216138, I28044, I54856, I104862, I143706, I172501, I234766, I28043, I54857, I104861, I143707, I172500, I14392, I30249, I54056, I105405, I14393, I30248, I54057, I105406, I23845, I46866, I75553, I120658, I23846, I46867, I75552, I120657, I37145, I89333, I135046, I37146, I89332, I135047, I30542, I56169, I84145, I131030, I30543, I56168, I84144, I131029, I37859, I67541, I89543, I137191, I37858, I67540, I89544, I44778, I74137, I98767, I139357, I44777, I74136, I98766, I139358, I23231, I44208, I76494, I23232, I44207, I76493, I24249, I47780, I74852, I120668, I157191, I204481, I24247, I47781, I74851, I120667, I157192, I204485, I49181, I80039, I122030, I158102, I49180, I80040, I122031, I158101, I31239, I30618, I57376, I90317, I30617, I57378, I90316, I133027, I12267, I49828, I15994, I32870, I32868, I57770, I111622, I34009, I34007, I62855, I111698, I20142, I36178, I62314, I90231, I229142, I246870, I261923, I300744, I370601, I460843, I248875, I263858, I297850, I234915, I251326, I270406, I305145, I371756, I255985, I274093, I337982, I292598, I391163, I302553, I321201, I282642, I303247, I325230, I362228, I414950, I879552, I1196891, I949029, I881980, I881981, I1025881, I1225896, I944327, I974943, I1174901, I981036, I1181388, I993285, I1193762, I1193770, I992208, I1061844, I1259263, I1058589, I1154769, I1157199, I1295274, I56699, I108755, I145818, I56700, I108756, I145819, I38569, I116025, I38570, I66498, I116024, I919238, I1058029, I19155, I41119, I67795, I116001, I153203, I189612, I19156, I41118, I67796, I116000, I153204, I189610, I19258, I40565, I66961, I116246, I153190, I189587, I19259, I40566, I66960, I116247, I153191, I189582, I25026, I51202, I76799, I25025, I51201, I76800, I120217, I25013, I52201, I80844, I119498, I157857, I196517, I25012, I52202, I80843, I119497, I157858, I196520, I943663, I1262194, I1262195, I28502, I56093, I84086, I131130, I160288, I204429, I28501, I56095, I84085, I131129, I160289, I204433, I55901, I90192, I129095, I30220, I38607, I73728, I97398, I137090, I38606, I73729, I97399, I137089, I38617, I69317, I97386, I136949, I38616, I69316, I97385, I136950, I38593, I73098, I98661, I136936, I38594, I73096, I98662, I136937, I40114, I72979, I93695, I93697, I137062, I40543, I72978, I93696, I137063, I956358, I1280292, I957065, I971748, I1320538, I984807, I1165491, I1320212, I996786, I1174125, I1045204, I1233828, I1345109, I1147596, I1457246, I1056281, I964899, I948826, I950885, I961497, I964917, I988748, I1188738, I1019265, I1037228, I1090449, I1159023, I1174356, I1182968, I984343, I957103, I1165397, I1334439, I958275, I1349605, I973656, I991861, I1211451, I1059028, I850396, I13152, I26923, I51120, I145486, I198299, I240222, I51121, I900932, I14768, I56879, I108019, I281467, I56878, I14861, I33976, I57065, I106120, I169076, I227049, I306083, I41158, I927511, I1255836, I1518314, I833977, I833978, I824980, I870102, I1237590, I1494796, I861838, I874809, I940645, I944650, I1304645, I973541, I1214909, I1214910, I992628, I1384597, I1462576, I1478584, I1478784, I1478765, I1490106, I1494601, I1514928, I1514753, I873878, I873879, I923444, I1265863, I915902, I1243833, I949924, I1274602, I959644, I959645, I1286970, I980386, I1347994, I986709, I1354011, I989129, I1371438, I1008726, I1177833, I1219607, I1358529, I1219675, I1359836, I1373547, I26642, I49305, I81763, I126241, I165881, I209047, I311334, I311329, I26641, I49304, I81764, I126242, I165882, I209048, I38022, I41845, I71034, I88469, I139452, I176995, I224500, I362561, I362567, I38023, I41844, I71035, I88468, I139457, I176996, I224504, I424991, I43812, I71185, I95039, I138987, I176986, I225690, I38953,

I95038, I138989, I176979, I992839, I1029891, I27782, I56740, I82985, I27783, I56739, I82984, I290819, I309724, I337991, I366387, I419431, I659911, I376929, I948808, I1272868, I874273, I1011188, I1480313, I1011216, I1480362, I1480363, I1036122, I1033265, I1480184, I1469940, I939835, I932015, I1267882, I892747, I1250826, I892735, I992757, I958916, I1006805, I1009372, I1032324, I1446679, I1461224, I1470229, I1469708, I1469854, I974164, I992246, I998049, I1031067, I1051051, I1049350, I1162191, I1162188, I14795, I30329, I14794, I30328, I15441, I55721, I110431, I146828, I15440, I55720, I110432, I146827, I18590, I18589, I40100, I70362, I116304, I153913, I40099, I70361, I116305, I153914, I27724, I54467, I82831, I27723, I54466, I82830, I31678, I55638, I86750, I86751, I31679, I55639, I30472, I57616, I30473, I57614, I32806, I58423, I86771, I86770, I109873, I135529, I32807, I135530, I44943, I46457, I76299, I76300, I76331, I99944, I146816, I168281, I168299, I44944, I46458, I76332, I99943, I100004, I146815, I168285, I54220, I81701, I81702, I106643, I54221, I106642, I953644, I979816, I979935, I986502, I1013527, I1014522, I1019224, I1024384, I1220298, I10818, I20035, I42634, I166653, I138224, I10819, I20034, I42635, I166645, I305995, I11398, I27158, I49245, I101138, I140907, I302425, I173885, I225441, I11399, I27157, I49244, I140906, I173881, I302426, I33576, I33575, I25255, I49288, I78226, I125200, I157296, I287097, I287104, I25254, I49287, I78227, I125201, I157295, I287092, I287095, I411284, I776224, I748891, I289561, I310187, I341921, I366942, I420368, I289659, I368415, I969812, I982856, I1028011, I1092240, I879209, I923016, I1415454, I939884, I1400194, I970020, I1335384, I980928, I1226508, I1355445, I992776, I1353787, I1020512, I1060030, I1387539, I1054709, I1387180, I1064839, I1387553, I1237740, I1438743, I14623, I14624, I14122, I29417, I54485, I105008, I1485506, I15347, I31215, I57582, I145868, I190823, I240574, I322851, I375049, I493357, I16627, I31837, I16377, I34098, I57188, I108796, I20370, I41122, I62359, I114885, I152986, I23350, I71123, I117987, I194712, I55704, I37848, I88979, I220326, I284251, I359023, I415628, I665351, I38467, I70113, I90037, I135218, I165814, I222009, I360825, I415160, I711042, I958094, I1280798, I982444, I977140, I977141, I1123765, I1304066, I1092329, I1120772, I1281547, I957994, I944155, I945601, I1122101, I1281566, I1015823, I1320573, I1047168, I1344400, I1029553, I1038941, I1261558, I1069428, I17176, I17177, I15564, I35356, I36310, I17639, I33704, I57433, I111737, I111738, I111747, I146765, I181028, I17205, I33703, I54322, I57474, I146764, I181022, I20487, I37834, I62817, I114943, I435634, I435637, I19546, I20488, I37833, I37865, I62816, I63908, I114944, I113409, I153373, I185557, I17906, I37399, I61949, I113410, I153372, I185554, I118807, I118806, I25171, I49319, I76848, I135509, I135508, I37340, I37341, I66830, I88931, I25444, I99698, I168411, I168410, I25445, I99697, I168409, I12355, I14379, I28531, I28573, I53879, I53895, I105300, I105335, I142878, I143006, I172291, I12356, I14380, I28530, I28572, I53878, I53896, I105299, I105334, I142879, I143005, I172285, I111824, I111825, I18896, I40945, I69267, I18895, I40946, I40946, I69268, I54603, I79439, I79456, I128194, I128221, I54593, I54602, I79438, I79457, I128192, I128222, I11203, I11101, I12459, I11806, I12294, I12313, I15366, I16150, I15821, I15556, I16090, I19021, I322059, I350824, I316547, I348299, I350743, I24827, I11566, I24826, I298714, I267862, I267860, I158205, I349457, I392942, I158204, I349467, I392932, I576772, I166068, I166067, I266209, I289592, I310244, I350052, I398577, I1153584, I267706, I285318, I302616, I346749, I400001, I291223, I323797, I347082, I295960, I316008, I342050, I342043, I367098, I423655, I300042, I322374, I322377, I342227, I342218, I369937, I423920, I342341, I358903, I370078, I341795, I358859, I395983, I342284, I353270, I364937, I392404, I342917, I369267, I347408, I348487, I360701, I373019, I398914, I10043, I28470, I40538, I105091, I135422, I165858, I226123, I10042, I28471, I40537, I105090, I135421, I226129, I15674, I15673, I14093, I36773, I57000, I104026, I143874, I143884, I173502, I235335, I14092, I15960, I36774, I104027, I143875, I143885, I173508, I235341, I14561, I16523, I30779, I31696, I60900, I60917, I109772, I110025, I14560, I16524, I30780, I31695, I60899, I60916, I109773, I110026, I16666, I35969, I57297, I113517, I16667, I35968, I57293, I113516, I19658, I20681, I38877, I38876, I41256, I49328, I76478, I76514, I99451, I100663, I137874, I138316, I41255, I49329, I76477, I76515, I99450, I100662, I138317, I367563, I379689, I14524, I31719, I31721, I54067, I105175, I144521, I14525, I31720, I54066, I105174, I144522, I14848, I33201, I107814, I144139, I14849, I33200, I107815, I144138, I16133, I38359, I62810, I113652, I147375, I16134, I38358, I62809, I113653, I29381, I89166, I129393, I29380, I29382, I89165, I129392, I35211, I63218, I87821, I139222, I35209, I63219, I87822, I139220, I13454, I28116, I52767, I104734, I142686, I179768, I13455, I28067, I52768, I142687, I179765, I11633, I23890, I45177, I100807, I139471, I23891, I45178, I100806, I139472, I11335, I25061, I42804, I95116, I142699, I174687, I235735, I235736, I11334, I25062, I42805, I95117, I142700, I174688, I235731, I365080, I417185, I13436, I26605, I13437, I26604, I47810, I13550, I27222, I53717, I104262, I142725, I13551,

I14339, I29207, I54428, I109882, I14338, I29206, I54429, I109880, I14815, I29960, I55414, I106255, I29959, I55413, I106254, I15474, I31189, I55827, I109338, I15473, I31190, I55826, I109337, I264213, I285010, I330167, I401076, I278515, I336201, I308400, I308417, I12584, I28089, I104847, I142514, I12583, I28088, I53629, I142515, I14916, I32626, I57391, I109470, I14917, I32627, I57395, I14224, I14223, I14178, I29637, I29636, I54627, I104632, I143203, I14176, I54628, I104631, I143202, I17816, I39406, I17815, I39407, I150902, I25113, I51635, I25112, I51636, I31702, I56062, I84304, I129029, I159958, I208651, I31701, I56063, I84305, I129028, I159959, I208656, I942799, I926793, I1246441, I929121, I1266559, I1366966, I915810, I1222159, I1271678, I1242424, I839474, I1101652, I1170869, I1475753, I851352, I1170887, I1472512, I887923, I1116406, I1229464, I935952, I1264670, I1523003, I974280, I974714, I1331108, I1018387, I1072748, I1324187, I1116063, I916365, I1348108, I874879, I1252024, I892759, I1256135, I899030, I900796, I1284408, I916399, I1304804, I917090, I925573, I930689, I944772, I945134, I951181, I946980, I1167318, I1299334, I947540, I1304737, I1348596, I986205, I987768, I992584, I1349784, I997533, I1381875, I998437, I998432, I1352191, I1008547, I1346152, I1031685, I1040036, I1040031, I1175356, I1175362, I1239535, I1240482, I1398870, I1275415, I1428762, I1327456, I1340855, I1056183, I1092019, I1231978, I982345, I1332317, I923853, I893552, I1500470, I1043570, I1486832, I874865, I1491890, I880643, I1192854, I1475933, I884964, I1209877, I1476589, I1252687, I1515308, I921879, I1256397, I1182315, I1332469, I1016136, I1332450, I1021968, I1022057, I1023542, I1335865, I1344417, I1044538, I1046901, I1360551, I1045984, I1227239, I1360295, I1046736, I1226810, I1343715, I1048378, I1343754, I1291638, I1428390, I1467526

Test set

The *Image Data ID*s of the 489 images used in the test set, which were also utilized for computing the metrics reported in Table 1 and Table 2, are listed below:

I831065, I989320, I1270020, I40503, I71097, I108155, I135395, I40502, I71096, I108154, I135396, I166844, I223895, I286514, I361615, I418013, I495940, I878146, I1255412, I938767, I1021530, I1252848, I912447, I1235535, I944379, I1423364, I11606, I11605, I11604, I25942, I45703, I103935, I25943, I45704, I103934, I17681, I19615, I36454, I38248, I66062, I66117, I113295, I116286, I152889, I217942, I17680, I36453, I66061, I113294, I116285, I217939, I17303, I39068, I66318, I113282, I152601, I214837, I269135, I17304, I39067, I66319, I113281, I152602, I214829, I269133, I25645, I53858, I25644, I53859, I17377, I47327, I17378, I47326, I23677, I47614, I78213, I121918, I23676, I47615, I78214, I121917, I258598, I272405, I302032, I340027, I393214, I9113, I17285, I129050, I164313, I210189, I9114, I30848, I85163, I129051, I164312, I210186, I10027, I24232, I45146, I10028, I24233, I45147, I37508, I37509, I40118, I47669, I74384, I74393, I40119, I47670, I74385, I74394, I974757, I1157071, I1325568, I1029029, I1346240, I1029013, I282010, I310437, I337398, I363615, I416762, I1041482, I1193331, I1154285, I300090, I314502, I343937, I368926, I33925, I55127, I111582, I145417, I153316, I191679, I239620, I371712, I15298, I33924, I55126, I111581, I145411, I153315, I239621, I951357, I966803, I1166993, I1037958, I1241179, I1490997, I1426128, I1190066, I1413030, I1151022, I1445758, I268927, I297175, I321443, I348191, I401405, I557380, I655561, I301491, I327812, I354833, I403915, I594111, I360312, I315851, I338808, I355328, I383448, I440554, I748885, I1018201, I1325857, I1021826, I12979, I27526, I50702, I12978, I27527, I50703, I50701, I31784, I58931, I86979, I112427, I31785, I58932, I86977, I86978, I112428, I1220921, I947480, I1264016, I1114881, I1296792, I1431781, I41527, I71261, I93875, I136668, I41528, I71260, I93876, I136669, I1295347, I1003706, I1186714, I1368078, I1016587, I1453675, I23213, I47251, I73289, I118226, I156289, I193378, I23212, I47250, I73290, I118225, I156288, I193386, I24406, I54638, I82864, I134330, I159681, I24404, I54637, I82865, I134331, I159682, I1147981, I19138, I40525, I67585, I115791, I19139, I40524, I67584, I115792, I19175, I65629, I117161, I19174, I65630, I117162, I24976, I24977, I25427, I50106, I77837, I120711, I158052, I196662, I25428, I50098, I77836, I120712, I158051, I941811, I30304, I56482, I84100, I129468, I160297, I204411, I30305, I56483, I84099, I129467, I160298, I204415, I955473, I1235084, I959742, I1129455, I1176653, I930417, I1415399, I916492, I1116728, I1403675, I13802, I29683, I55194, I106666, I146725, I894404, I971779, I1190623,

I998806, I1017725, I1196850, I1342389, I1514331, I944422, I1267895, I1003294, I1350223, I1047860, I357480, I372258, I389298, I415186, I1116451, I1189749, I1263792, I935436, I1477866, I1008180, I908698, I1070417, I1303143, I1378326, I983797, I1031290, I1469766, I947589, I1182766, I992286, I1079630, I19567, I36787, I68345, I126004, I19568, I36786, I68346, I126005, I40600, I41448, I41449, I70013, I106320, I166442, I166487, I138492, I40599, I70014, I106321, I166447, I166493, I138493, I1002458, I1024116, I10146, I20452, I41567, I10147, I20453, I41566, I387090, I925543, I1214021, I1011824, I1344946, I1391580, I1078739, I969402, I1296519, I1176879, I1477605, I17185, I25775, I119020, I119022, I119021, I25774, I49063, I77192, I16911, I17712, I33133, I33158, I58088, I111144, I111565, I147629, I147645, I181714, I17711, I33132, I33159, I58069, I111143, I111564, I147646, I181720, I23787, I44618, I77108, I119425, I23788, I44619, I77109, I119424, I45060, I78251, I142385, I45061, I78250, I12242, I12438, I1363593, I20543, I76696, I99797, I118284, I155002, I334134, I317119, I353803, I306383, I335241, I352395, I376255, I358772, I385031, I381316, I12195, I26800, I12194, I26801, I14838, I33751, I108387, I145531, I14837, I33752, I59844, I108386, I145530, I342517, I12028, I27894, I53593, I102444, I142415, I12030, I27895, I53594, I102445, I142416, I23112, I23113, I77119, I136029, I23111, I77118, I136028, I29268, I131842, I29269, I56099, I845577, I1152079, I1471354, I883190, I961824, I1136571, I1299107, I1018186, I1511356, I970943, I892771, I1280182, I930626, I983641, I992613, I1351301, I1181679, I1343923, I1444117, I1327480, I1037531, I1225971, I1170562, I1333802, I1481726, I1010650, I1014117, I1336238, I1486321

Supplementary Tables

				Real/Fake			Fake/Fake		
emb_{dim}	z_{dim}	λ_{cls}	AUC_{val}	FID	KID	SSIM	FID	KID	SSIM
2	100	2	0.7606	51.6823	0.0527	0.6002	0.0000	0.0000	0.6133
2	100	3	0.7553	48.2143	0.0461	0.6007	0.0000	0.0000	0.6131
2	100	4	0.7412	52.1503	0.0532	0.6062	0.0000	0.0000	0.6243
2	100	5	0.7632	48.7888	0.0492	0.6050	0.0000	0.0000	0.6203
2	100	6	0.7670	53.7360	0.0554	0.6055	0.0000	0.0000	0.6224
2	300	2	0.7615	52.0580	0.0533	0.6036	0.0000	0.0000	0.6197
2	300	3	0.7290	50.6333	0.0491	0.6024	0.0000	0.0000	0.6176
2	300	4	0.7657	52.3209	0.0525	0.6077	0.0000	0.0000	0.6286
2	300	5	0.7810	47.1469	0.0460	0.6008	0.0000	0.0000	0.6168
2	300	6	0.7509	50.9047	0.0491	0.6038	0.0000	0.0000	0.6221
2	512	2	0.7371	44.6461	0.0426	0.6052	0.0000	0.0000	0.6291
2	512	3	0.7741	50.9875	0.0494	0.6035	0.0000	0.0000	0.6220
2	512	4	0.7823	53.0211	0.0553	0.6065	0.0000	0.0000	0.6279
2	512	5	0.7631	55.0992	0.0575	0.6038	0.0000	0.0000	0.6239
2	512	6	0.7844	51.7242	0.0531	0.5991	0.0000	0.0000	0.6124
3	100	2	0.7590	54.8454	0.0578	0.5999	0.0000	0.0000	0.6122
3	100	3	0.7803	54.5463	0.0545	0.5958	0.0000	0.0000	0.6085
3	100	4	0.7681	48.3968	0.0464	0.5994	0.0000	0.0000	0.6128
3	100	5	0.7586	49.1977	0.0475	0.6014	0.0000	0.0000	0.6156
3	100	6	0.7723	52.8996	0.0545	0.6022	0.0000	0.0000	0.6184
3	300	2	0.7509	52.5197	0.0541	0.6050	0.0000	0.0000	0.6248
3	300	3	0.7679	59.2738	0.0635	0.6032	0.0000	0.0000	0.6232
3	300	4	0.7515	50.7141	0.0505	0.6008	0.0000	0.0000	0.6200
3	300	5	0.7889	57.8929	0.0590	0.6039	0.0000	0.0000	0.6258
3	300	6	0.7839	54.2160	0.0548	0.6017	0.0000	0.0000	0.6209
3	512	2	0.7549	53.4711	0.0530	0.6009	0.0000	0.0000	0.6174
3	512	3	0.7513	57.6130	0.0587	0.6052	0.0000	0.0000	0.6269
**3**	**512**	**4**	**0.7921**	**50.2808**	**0.0483**	**0.6104**	**0.0000**	**0.0000**	**0.6381**
3	512	5	0.7609	51.9038	0.0512	0.6019	0.0000	0.0000	0.6199
3	512	6	0.7643	59.4752	0.0605	0.6010	0.0000	0.0000	0.6166
4	100	2	0.7493	51.7196	0.0521	0.6018	0.0000	0.0000	0.6192
4	100	3	0.7741	48.6746	0.0456	0.6036	0.0000	0.0000	0.6205
4	100	4	0.7612	51.2276	0.0513	0.6028	0.0000	0.0000	0.6248
4	100	5	0.7798	49.2203	0.0479	0.6007	0.0000	0.0000	0.6178
4	100	6	0.7809	49.2994	0.0491	0.6008	0.0000	0.0000	0.6145
4	300	2	0.7746	49.2603	0.0492	0.6069	0.0000	0.0000	0.6257
4	300	3	-	-	-	-	-	-	-
4	300	4	0.7622	55.1574	0.0585	0.6024	0.0000	0.0000	0.6160
4	300	5	0.7624	53.0900	0.0516	0.6028	0.0000	0.0000	0.6191
4	300	6	0.7600	49.2006	0.0495	0.6030	0.0000	0.0000	0.6226
4	512	2	0.7555	52.8883	0.0537	0.6003	0.0000	0.0000	0.6189
4	512	3	0.7785	52.8223	0.0530	0.6029	0.0000	0.0000	0.6196
4	512	4	0.7854	49.0509	0.0476	0.6060	0.0000	0.0000	0.6294
4	512	5	0.7603	54.5842	0.0546	0.6024	0.0000	0.0000	0.6200
4	512	6	0.7714	54.9192	0.0570	0.6030	0.0000	0.0000	0.6260
5	100	2	0.7532	49.6793	0.0488	0.6046	0.0000	0.0000	0.6225
5	100	3	0.7635	48.7930	0.0493	0.6052	0.0000	0.0000	0.6230
5	100	4	0.7642	52.1934	0.0527	0.6036	0.0000	0.0000	0.6223
5	100	5	0.7620	56.2559	0.0569	0.5998	0.0000	0.0000	0.6150
5	100	6	0.7583	58.1718	0.0622	0.5973	0.0000	0.0000	0.6137
5	300	2	0.7516	50.4636	0.0523	0.5985	0.0000	0.0000	0.6113
5	300	3	0.7721	56.4171	0.0572	0.6001	0.0000	0.0000	0.6147
5	300	4	0.7748	53.7012	0.0550	0.6002	0.0000	0.0000	0.6197
5	300	5	0.7566	46.9046	0.0453	0.6035	0.0000	0.0000	0.6280

5	300	6	0.7903	60.5472	0.0635	0.6017	0.0000	0.0000	0.6197
5	512	2	0.7578	49.5425	0.0500	0.6072	0.0000	0.0000	0.6297
5	512	3	0.7589	52.0961	0.0495	0.6047	0.0000	0.0000	0.6246
5	512	4	0.7637	54.4425	0.0557	0.6050	0.0000	0.0000	0.6229
5	512	5	0.7795	54.1717	0.0542	0.6056	0.0000	0.0000	0.6263
5	512	6	0.7768	54.2638	0.0532	0.6010	0.0000	0.0000	0.6189
6	100	2	0.7312	48.4318	0.0485	0.6038	0.0000	0.0000	0.6202
6	100	3	0.7496	52.1170	0.0513	0.6027	0.0000	0.0000	0.6217
6	100	4	0.7603	50.0154	0.0486	0.6020	0.0000	0.0000	0.6197
6	100	5	0.7579	48.4102	0.0475	0.6013	0.0000	0.0000	0.6168
6	100	6	0.7535	46.9695	0.0449	0.6026	0.0000	0.0000	0.6199
6	300	2	0.7434	53.4041	0.0553	0.6027	0.0000	0.0000	0.6200
6	300	3	0.7726	53.4228	0.0526	0.6040	0.0000	0.0000	0.6308
6	300	4	-	-	-	-	-	-	-
6	300	5	0.7405	56.0304	0.0563	0.6057	0.0000	0.0000	0.6281
6	300	6	0.7629	53.3076	0.0534	0.6030	0.0000	0.0000	0.6220
6	512	2	0.7529	50.0542	0.0505	0.6039	0.0000	0.0000	0.6246
6	512	3	0.7320	54.9505	0.0578	0.6009	0.0000	0.0000	0.6176
6	512	4	0.7728	52.4141	0.0523	0.6073	0.0000	0.0000	0.6269
6	512	5	0.7754	48.5341	0.0465	0.6087	0.0000	0.0000	0.6328
6	512	6	0.7843	56.1221	0.0589	0.6012	0.0000	0.0000	0.6252
7	100	2	0.7670	52.9178	0.0534	0.6064	0.0000	0.0000	0.6259
7	100	3	0.7570	60.9971	0.0630	0.6009	0.0000	0.0000	0.6147
7	100	4	0.7868	47.1406	0.0436	0.6036	0.0000	0.0000	0.6225
7	100	5	0.7797	45.2168	0.0408	0.6059	0.0000	0.0000	0.6303
7	100	6	0.7523	50.6350	0.0497	0.6022	0.0000	0.0000	0.6176
7	300	2	0.7702	55.6051	0.0588	0.6013	0.0000	0.0000	0.6223
7	300	3	0.7534	48.8117	0.0476	0.6058	0.0000	0.0000	0.6254
7	300	4	0.7512	47.8945	0.0475	0.6071	0.0000	0.0000	0.6317
7	300	5	0.7530	51.9560	0.0525	0.6008	0.0000	0.0000	0.6146
7	300	6	0.7508	59.8121	0.0632	0.5974	0.0000	0.0000	0.6116
7	512	2	0.7798	52.7256	0.0525	0.6039	0.0000	0.0000	0.6234
7	512	3	0.7473	53.7119	0.0563	0.5991	0.0000	0.0000	0.6130
7	512	4	0.7655	55.9915	0.0588	0.6041	0.0000	0.0000	0.6192
7	512	5	0.7709	45.3785	0.0428	0.6022	0.0000	0.0000	0.6211
7	512	6	0.7777	67.5633	0.0719	0.5959	0.0000	0.0000	0.6105
8	100	2	0.7621	56.2211	0.0586	0.6007	0.0000	0.0000	0.6141
8	100	3	0.7746	48.7522	0.0475	0.6013	0.0000	0.0000	0.6130
8	100	4	0.7576	60.8971	0.0646	0.6014	0.0000	0.0000	0.6148
8	100	5	0.7910	48.3032	0.0479	0.6050	0.0000	0.0000	0.6214
8	100	6	0.7633	52.8528	0.0528	0.5988	0.0000	0.0000	0.6142
8	300	2	0.7657	52.3451	0.0518	0.6025	0.0000	0.0000	0.6181
8	300	3	0.7904	51.1367	0.0508	0.6066	0.0000	0.0000	0.6257
8	300	4	0.7572	54.0217	0.0552	0.6049	0.0000	0.0000	0.6297
8	300	5	0.7848	58.8569	0.0633	0.6035	0.0000	0.0000	0.6213
8	300	6	0.7660	52.3718	0.0524	0.6020	0.0000	0.0000	0.6197
8	512	2	0.7717	54.2751	0.0552	0.6014	0.0000	0.0000	0.6180
8	512	3	0.7434	56.7477	0.0580	0.6012	0.0000	0.0000	0.6175
8	512	4	0.7413	52.9178	0.0556	0.6071	0.0000	0.0000	0.6319
8	512	5	0.7742	52.4212	0.0536	0.6057	0.0000	0.0000	0.6302
8	512	6	0.7474	57.5165	0.0595	0.6044	0.0000	0.0000	0.6257

Supplementary Table 1: Results of the gird search for different combinations of the hyperparameters $\mathbf{emb_{dim}}$ (number of elements in which embed the class, to provide as an input to the generator), $\mathbf{z_{dim}}$ (dimension of the latent vector, input of the generator) and $\boldsymbol{\lambda_{cls}}$ (weight of the cross entropy in the training loss of the generator and discriminator). To evaluate the classification performance, we considered the Area Under the ROC Curve (AUC) measured on the validation set. To quantitatively assess the images generated by the generator, we considered the Frechét Inception Distance (FID), the Kernel Inception Distance (KID), and the Structural Similarity Index Measure (SSIM). The metrics in the section "Real/Fake" are computed by comparing images of the real distribution

(i.e., the test set) with images of the synthetic distribution (i.e., generated by the generator). These results must be compared with the similarity metrics computed between images of the fake distribution itself (see “Fake/Fake”) and of the real distribution itself (see Table 1). The empty lines refer to the hyperparameter configurations which did not reach convergence during the training process. The line underlined represents the performance of the final model that we selected as the best model.

Supplementary Figures

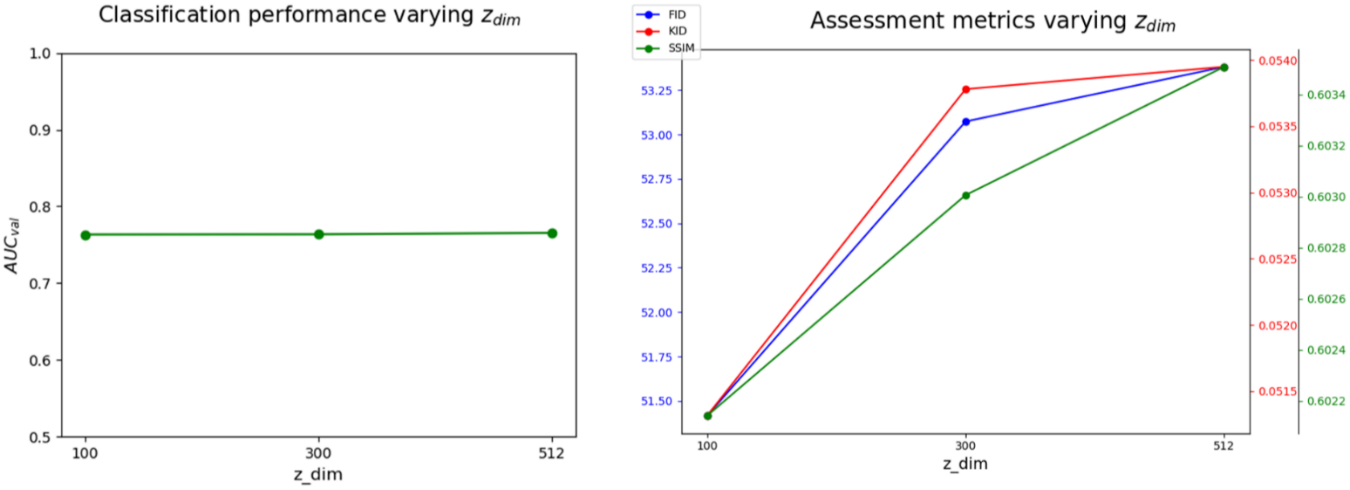

Supplementary Figure 1 Classification performance (left) and assessment metrics (right) averaged across all the results of the grid search for values of z_{dim} (dimension of *z*, the random vector input of the generator) of {100, 300, 512}. Note that each metric has a different scale, mapped by colour.

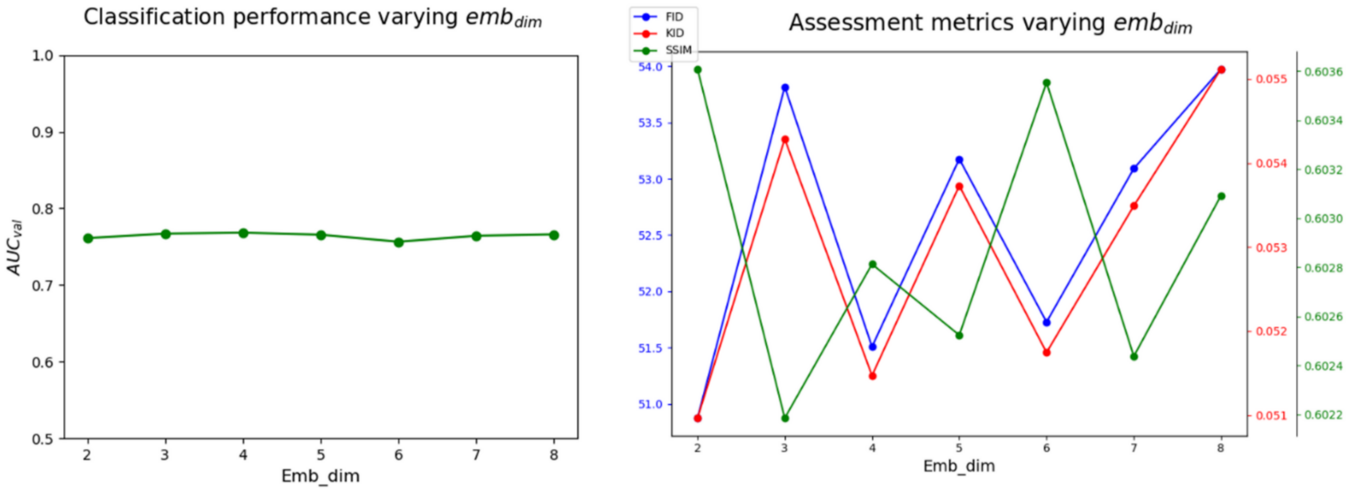

Supplementary Figure 2 Classification performance (left) and assessment metrics (right) averaged across all the results of the grid search for values of emb_{dim} (dimension of the embedding of the image class, to provide as input of the generator) of {2, 3, 4, 5, 6, 7, 8}. Note that each metric has a different scale, mapped by colour.

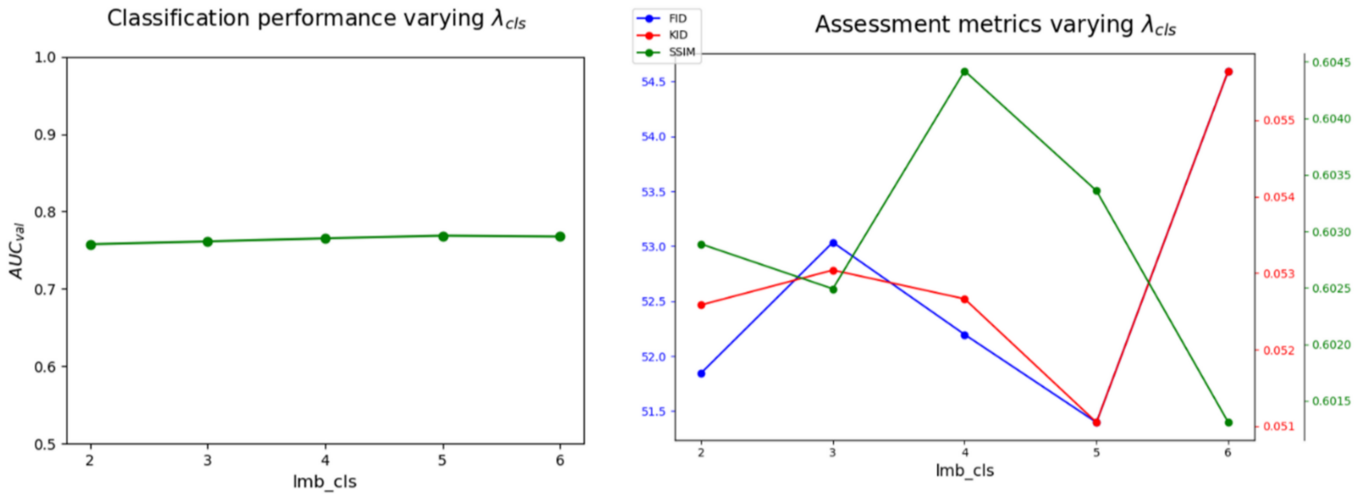

Supplementary Figure 3 Classification performance (left) and assessment metrics (right) averaged across all the results of the grid search for values of λ_{cls} (weight of the cross entropy in the loss function optimized during the training) of {2, 3, 4, 5, 6}. Note that each metric has a different scale, mapped by colour.
